# Synthesis, radiosynthesis, in vitro and first in vivo evaluation of a new matrix metalloproteinase inhibitor based on γ-fluorinated α-sulfonylaminohydroxamic acid

**DOI:** 10.1186/s41181-018-0045-0

**Published:** 2018-07-27

**Authors:** Verena Hugenberg, Malte Behrends, Stefan Wagner, Sven Hermann, Michael Schäfers, Hartmuth C. Kolb, Katrin Szardenings, Joseph C. Walsh, Luis F. Gomez, Klaus Kopka, Günter Haufe

**Affiliations:** 1 European Institute for Molecular Imaging, University of Münster, Waldeyerstr. 15, D-48149 Münster, Germany; 2 Department of Nuclear Medicine, University Hospital Münster, Albert-Schweitzer-Campus 1, Building A1, D-48149 Münster, Germany; 3 Organisch-Chemisches Institut, Westfälische Wilhelms-Universität Münster, Corrensstraße 40, D-48149 Münster, Germany; 4 Cells in Motion’ Cluster of Excellence, University of Münster, Waldeyerstr. 15, D-48149 Münster, Germany; 5 Siemens Medical Solutions USA, Inc., 6140 Bristol Parkway, Culver City, California, 90230 USA; 6 Present Address: Institute for Radiology, Nuclear Medicine and Molecular Imaging, Heart and Diabetes Center North Rhine Westphalia, University Hospital, Ruhr University Bochum, Georgstraße 11, D-32545 Bad Oeynhausen, Germany; 7 Present Address: German Cancer Research Center (dkfz), Division of Radiopharmaceutical Chemistry, Im Neuenheimer Feld 280, D-69120 Heidelberg, Germany

**Keywords:** Matrix metalloproteinase inhibitors, CGS 27023A analogues, In vitro assay, Amino hydroxamic acid, Fluorine; radiotracer, In vivo biodistribution

## Abstract

**Background:**

To study MMP activity in vivo in disease, several radiolabeled MMP inhibitors functioning as radiotracers have been evaluated by means of SPECT and PET. Unfortunately, most of them suffer from metabolic instability, mainly hepatobiliary clearance and insufficient target binding. The introduction of a fluorine atom into MMPIs could contribute to target binding, enhance the metabolic stability and might shift the clearance towards more renal elimination. Recently developed α-sulfonylaminohydroxamic acid based γ-fluorinated inhibitors of MMP-2 and -9 provide promising fluorine interactions with the enzyme active site and high MMP inhibition potencies. The aim of this study is the (radio)synthesis of a γ-fluorinated MMP-2 and -9 inhibitor to evaluate its potential as a radiotracer to image MMP activity in vivo.

**Results:**

Two new fluorine-containing, enantiomerically pure inhibitors for MMP-2 and -9 were synthesized in a six step sequence. Both enantiomers exhibited equal inhibition potencies in the low nanomolar and subnanomolar range. Log*D* value indicated better water solubility compared to the CGS 25966 based analog. The most potent inhibitor was successfully radiofluorinated. In vivo biodistribution in wild type mice revealed predominantly hepatobiliary clearance. Two major radioactive metabolites were found in different organs. Defluorination of the radiotracer was not observed.

**Conclusion:**

(Radio)synthesis of a CGS based γ-fluorinated MMP inhibitor was successfully accomplished. The (*S*)-enantiomer, which normally shows no biological activity, also exhibited high MMP inhibition potencies, which may be attributed to additional interactions of fluorine with enzyme’s active site. Despite higher hydrophilicity no significant differences in the clearance characteristics compared to non-fluorinated MMPIs was observed. Metabolically stabilizing effect of the fluorine was not monitored in vivo in wild type mice.

**Electronic supplementary material:**

The online version of this article (10.1186/s41181-018-0045-0) contains supplementary material, which is available to authorized users.

## Background

**Fig. 1:**
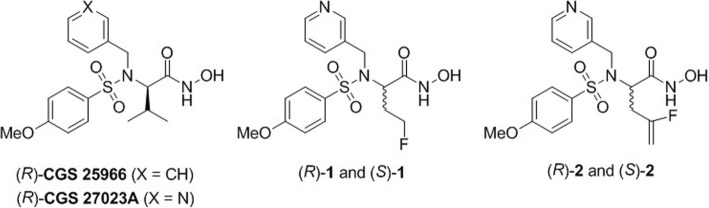
CGS lead structures and γ-fluorinated α-sulfonylaminohydroxamic acids based MMP inhibitors **1** und **2**

Matrix metalloproteinases (MMPs) are a family of structurally and functionally related zinc and calcium-dependent endopeptidases, responsible for the degradation and reconstruction of protein components within the extracellular matrix (ECM). They are involved in many physiological as well as pathological processes (Whittaker et al. 1999; Woessner and Nagase, 2000). Upregulated levels of MMPs are associated with various pathologies like inflammation, atherosclerosis (Brauer. 2006; Rai and Agrawal, 2017), and tumor progression (Sternlicht. 2001; Fingleton. 2006; Kessenbrock et al. 2010). MMP-2 and MMP-9 for example are capable to degrade type IV collagen, the most abundant component of the basement membrane. Degradation of the basement membrane allows cancer cells to migrate out of the primary tumor to form metastases. Upregulated expression of the gelatinases MMP-2 and MMP-9 directly correlates to an increased proliferation of tumor cells and tumor growth (Zhong et al. 2018). Especially for the diagnosis of breast cancer (Ren et al. 2015), non-small lung cancer (Gong et al. 2016) and ovarian endothelial carcinoma (Jia et al. 2017) the overexpression of MMP-2 and/or MMP-9 is used as a prognostic biomarker. Elevated levels of MMP-9 are found in rheumatoid arthritis (Gruber et al. 1996) and correlate with the progression of idiopathic atrial fibrillation (Li et al. 2014). Furthermore, overexpression of MMP-2 and -9 play a significant role in cardiovascular disease (Dimas et al. 2017) and in the development of aortic aneurysms (Rabkin. 2017). Therefore, many different MMP inhibitors (MMPIs) of the activated enzymes have been developed in recent years (Levin et al. 2017). Most of these MMPIs are broad spectrum inhibitors and gain their potency via the direct interaction with the catalytic Zn(II) ion in the active center of the enzyme (Jacobsen et al. 2010). Among different synthetic MMPIs, the sulfonamide-based hydroxamic acid derivatives CGS 25966 and CGS 27023A (Fig. 1) have been intensively investigated. Interestingly only the (*R*)*-*enantiomers of these hydroxamic acid derivatives are potent MMP inhibitors (Table 1), while the (*S*)-enantiomeric form demonstrated low or no inhibition potencies (Mac Pherson et al. 1997; Scozzafava et al. 1997) To the best of our knowledge, no IC_50_ values were published for the (*S*)-enantiomers.

**Table 1:** IC_50_ vcalues of CGS lead structures and γ-fluorinated MMP inhibitors **1** und **2** (Mac Pherson et al. 1997; Scozzafava et al. 2000; Behrends et al. 2015)

Compound	Configuration	IC_50_ values [nM]	
		MMP-2	MMP-9
**CGS 27023A**	*R*	20	8
**CGS 25966**	*R*	11	27
**1**	*R*	6.4	12.3
	*S*	32.8	3.0
**2**	*R*	9.3	8.3
	*S*	7.2	4.9

Based on lead structure CGS 27030A our group recently developed new potent fluorinated inhibitors **1** and **2** of MMP-2 and -9 (Table 1) (Behrends et al. 2015). Bearing a fluorine substituent in the γ-position of the amino acid core, these compounds seem to provide an additional possibility for the interaction with the active site of MMP-2 and MMP-9 and thereby, reducing the need of the stereocenter. In contrast to the lead compound CGS 27030A, where only the (*R*)-enantiomer shows inhibition potency for MMP-2 and -9, both enantiomers of the fluorinated hydroxamates **1** and **2** are potent inhibitors (Fig. 1). Modeling studies suggest that the γ-fluorine atom in the core of α-aminosulfonyl hydroxamic acid residues appear to influence the relative potencies via specific inhibitor-peptidase interactions, including short fluorine-hydrogen contacts, within the enzyme’s subpockets (Behrends et al. 2015).

To study MMP activity in vivo in disease, radiolabeled MMPIs functioning as radiotracers have been synthesized and evaluated by means of SPECT and PET. Unfortunately, most of the radiolabeled MMPIs suffer from insufficient metabolic instability, fast excretory elimination and insufficient target binding. Characteristically, the fast blood clearance and therefore insufficient bioavailability of radiolabeled MMPIs is based on a very efficient hepatobiliary tracer elimination (Matusiak et al. 2013).

The introduction of fluorine atom(s) into organic molecules is known to influence their pharmacokinetics, binding affinities and lipophilicity (O’Hagan. 2008; Purser et al. 2008; Begué and Bonnet-Delpon, 2008; Yamazaki et al. 2009; Gillis et al. 2015; Huchet et al. 2015; Meanwell, 2018). Thus, linkage of an additional fluorine-containing functional group in remote position to the zinc binding moiety might contribute to more efficient binding of the inhibitor in the active center of the enzyme (Behrends et al. 2015). Moreover, it could enhance the metabolic stability and might retard hepatobiliary elimination resulting in a shift of the clearance towards more renal elimination due to changes in hydrophilicity. In addition, a radiolabeled γ-fluorinated α-aminohydroxamic acid could possibly represent an effective radiotracer for imaging MMP-2 and -9 with improved pharmacokinetic properties and overall target binding affinities. Hence, the aim of this work is the synthesis of a γ-fluorinated MMP-2 and -9 inhibitor based on α-sulfonylaminohydroxamic acid with an additional fluorine-containing substituent allowing ^18^F-radiolabeling, and evaluation of its potential for the application as a radiotracer to image MMP activity in vivo.

## Methods

### General methods and chemistry

All chemicals, reagents and solvents for the synthesis of the compounds were analytical grade, purchased from commercial sources and used without further purification unless otherwise specified. All air and moisture-sensitive reactions were performed under argon atmosphere. Solvents were purified and dried analog to literature methods, where necessary. The melting points (mp) are uncorrected and were determined in capillary tubes on a Stuart Scientific SMP3 capillary melting point apparatus. Column chromatography was performed on Merck silica gel 60 (0.040–0.063 mm). Thin layer chromatography (TLC) was carried out on silica gel-coated polyester backed TLC plates (Polygram, SIL G/UV_254_, Macherey & Nagel) using solvent mixtures of cyclohexane (CH), ethyl acetate (EA) and methanol (MeOH). Compounds were visualized by UV light (254 nm). NMR spectra were recorded in CDCl_3_, CD_3_OH or DMSO-*d*_*6*_ on a Bruker ARX300, a Bruker DPX300 (^1^H NMR, 300 MHz, ^13^C NMR, 75 MHz, ^19^F NMR, 282 MHz), a Bruker AMX 400 (^1^H NMR, 400 MHz, ^13^C NMR, 100 MHz) and a Varian Unity plus 600 (^1^H NMR, 600 MHz, ^13^C NMR, 151 MHz) spectrometer. TMS (^1^H), CDCl_3_, DMSO-*d*_*6*_, CD_3_OD (^13^C) and CFCl_3_ (^19^F) were used as internal standards and all chemical shift values are reported in ppm (*δ*). Exact mass analyses were conducted on a Bruker MicroTof apparatus. The chemical and radiochemical purities of each new non-radioactive and radioactive compound were ≥ 95% and assessed by analytical gradient reversed-phase HPLC system **A**. HPLC system **A**: Two K-1800 pumps and an S-2500 UV detector (Herbert Knauer GmbH), a GabiStar γ-detector (Raytest Isotopenmessgeräte GmbH). The recorded data were processed by the ChromGate HPLC software (Herbert Knauer GmbH). The HPLC method **A1** started with a linear gradient from 10% to 90% CH_3_CN in water (0.1% TFA) over 9 min, followed by a linear gradient from 90% to 10% CH_3_CN in water (0.1% TFA) over 6 min, with a flow rate of 1 mL·min^− 1^.

*p*-(2-Fluoroethoxy)phenylsulfonyl chloride (**3a**) (Wagner et al. 2011), 2-[4-(chlorosulfonyl)phenoxy]ethyl-4-methylbenzenesulfonate (**3b**) (Wagner et al. 2011), *tert*-butyl (*S*)-2-amino-4-fluoropent-4-enecarboxylate ((*S*)-**4**) (Laue et al. 2000) and *tert*-butyl (*R*)-2-amino-4-fluoropent-4-enecarboxylate ((*S*)-**4**) (Laue et al. 2000) were synthesized following literature procedures. All animal experiments were conducted in accordance with local institutional guidelines for the care and use of laboratory animals.

### Preparation of MMPI (*S*)-**9a**

#### *Tert*-butyl (*S*)-4-fluoro-2-{[4-(2-fluoroethoxy)phenyl]sulfonamide}pent-4-enoate ((*S*)-**5a**)

To a solution of *tert*-butyl (*S*)-2-amino-4-fluoropent-4-enecarboxylate ((*S*)-**4**) (892 mg, 4.7 mmol) in pyridine *p*-(2-fluoroethoxy)-phenylsulfonylchloride (1130 mg, 4.7 mmol) was added under stirring at 0 °C. The reaction mixture was allowed to warm up to rt. and stirred for 40 h. Then the mixture was diluted with dichloromethane and the organic solution was washed with 0.5 N HCl, water (2 times each) and brine. After drying with magnesium sulfate the solvent was removed in vacuum. The crude product was passed through a short pad of silica gel and recrystallized from ethyl acetate/cyclohexane. The product was isolated as a colorless crystalline solid (1.18 g, 64%); mp 102 °C (EA/CH). ^1^H NMR (300 MHz, CDCl_3_): δ = 7.79 (dm, ^3^*J*_H,H_ = 9.0 Hz, Ar*H*, 2H,), 6.99 (dm, ^3^*J*_H,H_ = 9.0 Hz, Ar*H*, 2H), 5.37 (d, ^3^*J*_H,H_ = 9.0 Hz, N*H*, 1H), 4.77 (dm, ^2^*J*_H,F_ = 47.5 Hz, CH_2_F, 2H), 4.64 (dd, ^3^*J*_H,F_ = 17.1 Hz, ^2^*J*_H,H_ = 3.1 Hz, CF=C*H*_2_, H_*cis*_, 1H), 4.35 (dd, ^3^*J*_H,F_ = 49.3 Hz, ^2^*J*_H,H_ = 3.1 Hz, CF=C*H*_2_, H_*trans*_, 1H), 4.26 (dm, ^3^*J*_H,F_ = 27.7 Hz, C*H*_2_CH_2_F, 2H), 3.98 (dt, ^3^*J*_H,H_ = 8.8 Hz, ^3^*J*_H,H_ = 5.8 Hz, NHC*H*, 1H), 2.65 (d, ^3^*J*_H,H_ = 5.9 Hz, *NHCHCH*_2_, H_*A*_, 1H), 2.59 (dd, ^3^*J*_H,H_ = 5.7 Hz, ^3^*J*_H,H_ = 4.2 Hz, NHCHC*H*_2_, H_B_, 1H), 1.30 (s, C(C*H*_3_)_3_, 9H). ^13^C NMR (75 MHz, CDCl_3_): δ = 169.2 (*C*O), 161.8 (Ar*C*O), 160.9 (d, ^1^*J*_C,F_ = 256.9 Hz, CF), 131.9 (Ar*C*SO_2_), 129.5 (Ar*C*H), 114.7 (Ar*C*H), 94.1 (d, ^2^*J*_C,F_ = 18.8 Hz, CH_2_ = CF), 83.2 (*C*(CH_3_)_3_), 81.5 (d, ^1^*J*_C,F_ = 171.5 Hz, *C*H_2_F), 67.4 (dt, ^2^*J*_C,F_ = 20.4 Hz, *C*H_2_CH_2_F), 53.2 (d, ^3^*J*_C,F_ = 1.0 Hz, NH*C*H), 36.4 (dt, ^2^*J*_C,F_ = 27.6 Hz, NHCH*C*H_2_), 27.6 (C(*C*H_3_)_3_). ^19^F NMR (282 MHz, CDCl_3_): δ = − 96.1 (ddt, ^3^*J*_H,F_ = 49.3 Hz, ^3^*J*_H,F_ = 20.4 Hz, ^3^*J*_H,F_ = 17.2 Hz, CF), − 224.3 (tt, ^2^*J*_H,F_ = 47.3 Hz, ^3^*J*_H,F_ = 27.7 Hz, CH_2_F). Elemental analysis: C_17_H_23_F_2_NO_5_S (M = 391.43 g/mol), calcd. C 52.16, H 5.92, N 3.58; found C 52.64, H 6.24, N 3.55%. MS-ES(+)-EM: *m/z =* 414.1160 [(M + Na)^+^] calcd. For C_17_H_23_F_2_NO_5_SNa^+^: 414.1163.

#### *Tert*-butyl (*S*)-2-{[*N*-benzyl-4-(2-fluoroethoxy)phenyl]sulfonamide}-4-fluoropent-4-enoate ((*S*)-**6a**)

*Tert*-Butyl *N*-[*p-*(-2-fluoroethoxy)phenylsulfonyl]aminopent-4-enoate (830 mg, 2.12 mmol), dissolved in dimethylformamide (20 mL) was treated under stirring with 10 equivalents of potassium carbonate (2.93 g, 21.2 mmol). After 20 min benzyl bromide (363 mg, 2.12 mmol, 1 equiv) was added and the mixture was stirred at rt. for 40 h. Then water was added and the mixture was extracted with ethyl acetate (4 times). The combined organic extracts were washed with water (4 times), brine and dried with magnesium sulfate. After removal of the solvent the crude product was purified chromatographically (silica gel, CH/EA, 4:1). The product was obtained as a colorless yellowish viscos oil (800 mg, 78%). ^1^H NMR (300 MHz, CDCl_3_): δ = 7.82 (dm, ^3^*J*_H,H_ = 9.0 Hz, Ar*H*, 2H), 7.29 (m, 14-CH, 15-CH, Ar*H*, 5H), 6.98 (dm, ^3^*J*_H,H_ = 8.9 Hz, Ar*H*, 2H), 4.80 (dm, ^2^*J*_H,F_ = 47.3 Hz, CH_2_F, 2H), 4.65 (d, ^2^*J*_H,H_ = 16.0 Hz, NC*H*_2_, H_A_, 1H), 4.56 (t, ^3^*J*_H,H_ = 7.3 Hz, NC*H*, 1H), 4.53 (dd, ^3^*J*_H,F_ = 17.1 Hz, ^2^*J*_H,H_ = 3.0 Hz, CF=CH_2_, H_*cis*_, 1H), 4.29 (d, ^2^*J*_H,H_ = 15.7 Hz, NC*H*_2_, H_B_, 2H), 4.25 (dm, ^3^*J*_H,F_ = 27.8 Hz, C*H*_2_CH_2_F, 2H), 4.11 (dd, ^3^*J*_H,F_ = 49.9 Hz, ^2^*J*_H,H_ = 3.0 Hz, CF=C*H*_2_, H_*trans*_, 1H), 2.70 (dt, ^3^*J*_H,F_ = 15.3 Hz, ^3^*J*_H,H_ = 6.9 Hz, NCH_2_, H_A_, 1H), 2.46 (ddd, ^3^*J*_H,F_ = 20.0 Hz, ^2^*J*_H,H_ = 15.1 Hz, ^3^*J*_H,H_ = 7.6 Hz, NC*H*_2_, H_B_, 1H), 1.36 (s, C(CH_3_)_3_, 9H). ^13^C NMR (75 MHz, CDCl_3_): δ = 168.5 (CO), 161.9 (ds, ^1^*J*_C,F_ = 256.5 Hz, CF), 161.7 (Ar*C*O), 136.7 (Ar*C*CH_2_), 132.5 (Ar*C*SO_2_), 129.9 (Ar*C*H), 128.5 (Ar*C*H), 128.4 (Ar*C*H), 127.7 (Ar*C*H), 114.6 (Ar*C*H), 93.1 (dt, ^2^*J*_C,F_ = 19.2 Hz, CF=*C*H_2_), 82.6 (*C*(CH_3_)_3_), 81.5 (dt, ^1^*J*_C,F_ = 171.6 Hz, *C*H_2_F), 67.4 (dt, ^2^*J*_C,F_ = 20.2 Hz, *C*H_2_CH_2_F), 57.6 (N*C*H), 50.0 (N*C*H_2_), 34.1 (dt, ^2^*J*_C,F_ = 27.9 Hz, NCH*C*H_2_), 27.8 (C(*C*H_3_)_3_). ^19^F NMR (282 MHz, CD_3_CN): δ = − 97.7 (m, ^3^*J*_H,F_ = 49.8 Hz, ^3^*J*_H,F_ = 19.8 Hz, ^3^*J*_H,F_ = 16.4.0 Hz, CF), − 224.3 (tt, ^2^*J*_H,F_ = 47.3 Hz, ^3^*J*_H,F_ = 27.6 Hz, CH_2_F). MS-ES(+)-EM: *m/z =* 504.1613 [(M + Na)^+^] calcd. For C_24_H_29_F_2_NO_5_SNa^+^: 504.1632.

#### (*S*)-2-{[*N*-Benzyl-4-(2-fluoroethoxy)phenyl]sulfonamide}-4-fluoropent-4-enoic acid ((*S*)-**7a**)

In a dried YOUNG-tube *tert*-butyl *N-*benzyl-(*S*)-*N*-[*p*-(2-fluoroethoxy)phenyl-sulfonyl]-2-amino-4-fluoropent-4-enoate ((*S*)-**6a**) (400 mg, 0.94 mmol) was dissolved in dry dichloromethane (20 mL) under argon and treated with trifluoroacetic acid (20 mL). The YOUNG-tube was flushed with argon and sealed. The mixture was stirred at rt. for 3–4 h. Subsequently the reaction mixture was evaporated to dryness in vacuum. The residue was dissolved in chloroform (100 mL) and washed with an aqueous solution of citric acid and sodium bicarbonate (25 mL, pH ≈ 4). The aqueous phase was extracted with chloroform (4 × 30 mL) and the combined organic phases were dried with magnesium sulfate. After evaporation of the solvent the crude product was obtained as colorless, viscos oils, which in high vacuum solidified. The product was used for the next step without further purification (160 mg, 40%). ^1^H NMR (300 MHz, CD_3_CN): δ = 7.78 (dm, ^3^*J*_H,H_ = 9.1, Ar*H*, 2H), 7.29 (m, Ar*H*, Ar*H*, Ar*H*, 5H), 7.04 (dm, ^3^*J*_H,H_ = 9.0 Ar*H*, 2H,), 4.75 (dm, ^2^*J*_H,F_ = 47.7 Hz, CH_2_F, 2H), 4.65 (dd, ^3^*J*_H,H_ = 8.5, ^3^*J*_H,H_ = 6.0, NC*H*, 1H), 4.57 (d, ^2^*J*_H,H_ = 16.1, NC*H*_2_, H_A_, 1H), 4.52 (dd, ^3^*J*_H,F_ = 17.6, ^2^*J*_H,H_ = 3.0, CF=C*H*_2_, H_*cis*_, 1H), 4.32 (d, ^2^*J*_H,H_ = 16.1, NC*H*_2_, H_B_, 1H), 4.30 (dm, ^3^*J*_H,F_ = 29.4 Hz, CH_2_CH_2_F, 2H), 4.14 (dd, ^3^*J*_H,F_ = 51.8, ^2^*J*_H,H_ = 3.4, CF=C*H*_2_, H_*trans*_, 1H), 2.76 (ddd, ^3^*J*_H,F_ = 14.7, ^2^*J*_H,H_ = 13.7, ^3^*J*_H,H_ = 6.0, NC*H*_2_, H_A_, 1H), 2.47 (ddd, ^3^*J*_H,F_ = 22.2, ^2^*J*_H,H_ = 15.4, ^3^*J*_H,H_ = 8.5, NC*H*_2_, H_B_, 1H). ^13^C NMR (75 MHz, CD_3_CN): δ = 171.2 (*C*O), 163.2 (d, ^1^*J*_C,F_ = 254.7 Hz, CF), 163.1 (Ar*C*O), 138.2 (Ar*C*CH_2_), 133.0 (Ar*C*SO_2_), 131.0 (Ar*C*H), 129.6 (Ar*C*H), 129.3 (Ar*C*H), 128.7 (Ar*C*H), 115.7 (Ar*C*H), 93.8 (d, ^2^*J*_C,F_ = 18.9 Hz, CF=*C*H_2_), 83.1 (d, ^1^*J*_C,F_ = 167.5 Hz, *C*H_2_F), 68.8 (d, ^2^*J*_C,F_ = 19.2 Hz, *C*H_2_CH_2_F), 57.9 (N*C*H), 50.8 (N*C*H_2_), 34.2 (d, ^2^*J*_C,F_ = 28.0 Hz, NCH*C*H_2_). ^19^F NMR (282 MHz, CD_3_CN): δ = − 97.7 (dddd, ^3^*J*_H,F_ = 50.9 Hz, ^3^*J*_H,F_ = 22.1 Hz, ^3^*J*_H,F_ = 17.7 Hz, ^3^*J*_H,F_ = 13.3 Hz, CF), − 224.0 (tt, ^2^*J*_H,F_ = 47.7 Hz, ^3^*J*_H,F_ = 29.5 Hz, CH_2_F). MS-ES(+)-EM: *m/z =* 448.1009 [(M + Na)^+^] calcd. For C_20_H_31_F_2_NO_5_SNa^+^: 448.1006.

#### (2*S*)-2-[*N*-Benzyl-4-(2-fluoroethoxy)phenylsulfonamido]-4-fluoro-*N*-[(tetrahydro-2H-pyran-2-yl)oxy]pent-4-enamide ((*S*)-**8a**)

To a solution of (*S*)-2-{[*N*-benzyl-4-(2-fluoroethoxy)phenyl]sulfonamido}-4-fluoropent-4-enoic acid ((*S*)-**7a**) (100 mg, 0.235 mmol) in DMF (0.06 mmol/mL, 1 mL) 1-hydroxybenzotriazole hydrate (HOBT, 1.2 eq., 38 mg, 0.282 mmol), 4-methylmorpholine (NMM, 3.0 eq., 78 μL, 249.70 mmol), *O*-tetrahydro-2-*H*-pyran-2-yl-hydroxylamine (3.1 eq., 85 mg, 0.729 mmol and *N*-[(dimethylamino)-propyl]-*N*′-ethylcarbodiimide hydrochloride (EDC, 1.4 eq., 63 mg, 0.329 mmol) were added. After stirring overnight at room temperature the reaction mixture was diluted with water (20 mL) and extracted with ethyl acetate (3 × 5 mL). The combined organic phases were washed successively with water, 5% aqueous KHSO_4_, saturated aqueous NaHCO_3_ and brine, and dried over magnesium sulfate. After removing the solvent under reduced pressure column chromatographic purification (silica gel, CH/EA 2:1) yielded the THP-protected hydroxamic acid as a light brown wax (92 mg, 75%). ^1^H NMR (300 MHz, CDCl_3_): δ = 9.19 (s, NH, 1H), 9.15 (s, NH, 1H), 7.77 (d, ^3^*J*_H,H_ = 8.9 Hz, Ar*H*, 2H), 7.72 (d, ^3^*J*_H,H_ = 9.0 Hz, Ar*H*, 2H), 7.39–7.21 (m, Ar*H*, 5H), 6.98 (d, ^3^*J*_H,H_ = 9.0 Hz, Ar*H*, 2H), 6.97 (d, ^3^*J*_H,H_ = 9.0 Hz, Ar*H*, 2H), 4.92–4.88 (m, NHOC*H*O), 4.79 (dm, ^2^*J*_H,F_ = 47.4 Hz, C*H*_2_F, 2H), 4.66 (AB, d, ^2^*J*_H,H_ = 16.0 Hz, NC*H*_2_, 1H), 4.58–4.45 (m, NC*H*, NC*H*_2_, 2H), 4.43 (dd, CFC*H*_2_*,* H_*cis*_, ^3^*J*_H,F_ = 17.1 Hz, ^2^*J*_H,F_ = 3.0 Hz, 1H), 4.39 (dd, CFC*H*_2_*,* H_*cis*_, ^3^*J*_H,F_ = 17.1 Hz, ^2^*J*_H,F_ = 3.0 Hz, 1H), 4.27 (dm, ^3^*J*_H,F_ = 27.7 Hz, O-C*H*_2_CH_2_F, 2H), 4.10 (dd, CFC*H*_2_*,* H_*trans*_, ^3^*J*_H,F_ = 49.9 Hz, ^2^*J*_H,F_ = 3.1 Hz, 1H), 3.95–3.84 (m, NHOCHOC*H*_2_, 1H), 3.70–3.55 (m, NHOCHOC*H*_2_, 1H), 2.93–2.58 (AB, m, NCHC*H*_2_, 1H), 2.47–2.18 (AB, m, NCHC*H*_2_, 1H), 1.88–1.50 (m, THP-C*H*_2_, 6H). ^13^C NMR (75 MHz, CDCl_3_): δ = 165.8 (*C*ONH), 165.6 (*C*ONH), 162.0 (qAr*C*OCH_2_CH_2_F), 161.2 (d, ^1^*J*_C,F_ = 256.3 Hz, *C*FCH_2_), 161.2 (d, ^1^*J*_C,F_ = 256.0 Hz, *C*FCH_2_), 136.6 (qAr*C*CH_2_N), 136.5 (qAr*C*CH_2_N), 131.5 (qAr*C*SO_2_), 129.7 (Ar*C*H), 129.7 (Ar*C*H), 128.7 (Ar*C*H), 128.7 (Ar*C*H), 128.5 (Ar*C*H), 128.4 (Ar*C*H), 127.8 (Ar*C*H), 127.8 (Ar*C*H), 114.7 (Ar*C*H), 114.7 (Ar*C*H), 102.1 (NHO*C*HO), 101.7 (NHO*C*HO), 93.7 (d, ^2^*J*_C,F_ = 19.0 Hz, CF*C*H_2_), 93.6 (d, ^2^*J*_C,F_ = 18.9 Hz, CF*C*H_2_), 81.4 (^1^*J*_C,F_ = 171.6 Hz, OCH_2_*C*H_2_F), 67.4 (d, ^2^*J*_C,F_ = 20.5 Hz, O*C*H_2_CH_2_F), 62.0 (NHOCHO*C*H_2_), 54.7 (N*C*H), 54.5 (N*C*H), 48.6 (N*C*H_2_), 48.5 (N*C*H_2_), 32.3 (d, ^2^*J*_C,F_ = 27.6 Hz, NCH*C*H_2_CF), 32.0 (d, ^2^*J*_C,F_ = 26.9 Hz, NCH*C*H_2_CF), 27.7 (NHOCH*C*H_2_), 27.7 (NHOCH*C*H_2_), 24.9 (CH_2_), 18.2 (NHOCHCH_2_*C*H_2_). ^19^F NMR (282 MHz, CD_3_CN): δ = − 97.47 (m, CFCH_2_, 1F), − 97.72 (m, CFCH_2_, 1F), − 223.89 (tt, ^2^*J*_H,F_ = 47.3, ^3^*J*_H,F_ = 27.6 Hz, OCH_2_CH_2_F, 1F), − 223.91 (tt, ^2^*J*_H,F_ = 47.3, ^3^*J*_H,F_ = 27.6 Hz, OCH_2_CH_2_F, 1F) (two signal sets due to the presence of two stereoisomers). MS-ES-EM: *m/z* = 547.1683 [(M + Na)^+^] calcd for C_25_H_30_F_2_N_2_O_6_SNa^+^: 547.1685.

#### (*S*)-2-[*N*-Benzyl-4-(2-fluoroethoxy)phenylsulfonamido]-4-fluoro-*N*-hydroxypent-4-enamide ((*S*)-**9a**)

To a solution of (2*S*)-2-[*N*-benzyl-4-(2-fluoroethoxy)phenylsulfonamido]-4-fluoro-*N*-[(tetrahydro-2*H*-pyran-2-yl)oxy]pent-4-enamide ((*S*)-**8a**) (92 mg, 0.175 mmol) in 1,4-dioxane (0.5 mL/0.18 mmol) 4 N hydrochloric acid in 1,4-dioxane (4 eq.) and methanol (0.5 mL/0.18 mmol) were added and the reaction mixture was stirred at room temperature. The reaction progress was monitored by TLC (ethyl acetate). After complete conversion the reaction mixture was diluted with ethyl acetate (20 mL), washed with water (3 × 10 mL) and dried (MgSO_4_). The solvent was removed under reduced pressure and the product was purified by column chromatography (silica gel, CH/EA 2:1). The product was obtained as a light brown wax (33 mg, 43%). ^1^H NMR (400 MHz, CDCl_3_): δ = 9.19 (s, 1 OH), 7.72 (d, ^3^*J*_H,H_ = 8.9 Hz, Ar*H*, 2H), 7.35–7.25 (m, Ar*H*, 5H), 6.97 (d, ^3^*J*_H,H_ = 8.9 Hz, Ar*H*, 2H), 4.79 (dm, ^2^*J*_H,F_ = 47.3 Hz, C*H*_2_F, 2H), 4.54 (AB, d, ^2^*J*_H,H_ = 15.6 Hz, NC*H*_2_, 1H), 4.52 (m, NC*H*, 1H), 4.46 (AB, d, ^2^*J*_H,H_ = 15.7 Hz, NC*H*_2_, 1H), 4.38 (dd, CFC*H*_2_, H_*cis*_, ^3^*J*_H,F_ = 17.0 Hz, ^2^*J*_H,F_ = 2.9 Hz, 1H), 4.26 (dm, ^3^*J*_H,F_ = 27.6 Hz, CH_2_FC*H*_2_, 2H), 4.08 (dd, CFC*H*_2_*,* H_*trans*_, ^3^*J*_H,F_ = 49.7 Hz, ^2^*J*_H,F_ = 3.1 Hz, 1H), 2.85–2.69 (AB, m, NCHC*H*_2_, 1H), 2.38 (AB, m, NCHC*H*_2_, 1H). ^13^C NMR (101 MHz, CDCl_3_): δ = 166.4 (CONH), 162.1 (qAr*C*OCH_2_CH_2_F), 161.1 (d, ^1^*J*_C,F_ = 256.3 Hz, *C*FCH_2_), 136.1 (qAr*C*CH_2_N), 131.3 (qAr*C*SO_2_), 129.7 (Ar*C*H), 128.6 (Ar*C*H), 128.6 (Ar*C*H), 128.0 (Ar*C*H), 114.8 (Ar*C*H), 93.8 (d, ^2^*J*_C,F_ = 18.9 Hz, CF*C*H_2_), 81.4 (d, ^1^*J*_C,F_ = 171.7 Hz, OCH_2_*C*H_2_F), 67.4 (d, ^2^*J*_C,F_ = 20.4 Hz, O*C*H_2_CH_2_F), 54.2 (N*C*H), 48.7 (N*C*H_2_), 31.7 (d, ^2^*J*_C,F_ = 27.7 Hz, NCH*C*H_2_CF). ^19^F NMR (282 MHz, CDCl_3_): δ = − 97.58 (m, CFCH_2_, 1F), − 223.84 (tt, ^2^*J*_H,F_ = 47.3, ^3^*J*_H,F_ = 27.6 Hz, OCH_2_CH_2_F, 1F). MS-ES-EM: *m/z* = 463.1120 [(M + Na)^+^] calcd for C_20_H_22_F_2_N_2_O_5_SNa^+^: 463.1110. HPLC t_*R*_ = 9.05 min (100%).

### Radiochemistry

#### General methods

Radiofluorinations were carried out on a modified PET tracer radiosynthesizer (TRACERLab FxFDG, GE Healthcare). The recorded data were processed by the TRACERLab Fx software (GE Healthcare). Separation and purification of the radiosynthesized compounds were performed on the following semi-preparative radio-HPLC system **B** (λ = 254 nm): K-500 and K-501 pump, K-2000 UV detector (Herbert Knauer GmbH), NaI(TI) Scintibloc 51 SP51 γ-detector (Crismatec) and a Nucleosil 100–10 C18 column (5 μ, 250 × 8 mm). The recorded data were processed by the GINA Star software (Raytest Isotopenmessgeräte GmbH). The HPLC method **B1** started with a linear gradient from 30% to 70% CH_3_CN in water (0.1% TFA) over 30 min, holding for 5 min, followed by a linear gradient from 70% to 30% CH_3_CN in water (0.1% TFA) over 5 min, with a flow rate of 4 mL·min^− 1^. Radiochemical purities and specific activities were determined using the analytical radio-HPLC system **C** and HPLC method **C1**. HPLC System C: Two Smartline 1000 pumps and a Smartline UV detector 2500 (Herbert Knauer GmbH), a GabiStar γ-detector (Raytest Isotopenmessgeräte GmbH) and a Nucleosil 100–5 C-18 column (250 mm × 4.6 mm). The recorded data were processed by the GINA Star software (Raytest Isotopenmessgeräte GmbH). HPLC method **C1** started isocratic with 40% CH_3_CN in water (0.1% TFA) for 22 min, followed by a linear gradient from 40% to 90% CH_3_CN in water (0.1% TFA) over 4 min, followed by a linear gradient from 90% to 40% CH_3_CN in water (0.1% TFA) over 4 min, with a flow rate of 1 mL·min^− 1^. No-carrier-added aqueous [^18^F]fluoride was produced on a RDS 111e cyclotron (CTI-Siemens) by irradiation of a 1.2 mL water target using 10 MeV proton beams on 97.0% enriched [^18^O]water by the ^18^O(p,n)^18^F nuclear reaction.

#### (*S*)-2-[*N*-Benzyl-4-(2-[^18^F]fluoroethoxy)phenylsulfonamido]-4-fluoro-*N*-hydroxypent-4-enamide ((*S*)-[^18^F]**9a**)

In a computer controlled TRACERLab Fx_FDG_ synthesizer aqueous [^18^F]fluoride ions (1.0–5.0 GBq) from the cyclotron target were passed through an anion exchange resin (SepPak Light Waters Accell Plus QMA cartridge, preconditioned with 5 mL of 1 M K_2_CO_3_ and 10 mL of water for injection). [^18^F]Fluoride ions were eluted from the resin with a mixture of 40 μL of 1 M K_2_CO_3_, 200 μL of water for injection, and 800 μL of DNA-grade CH_3_CN containing 18 mg (48 μmol) of Kryptofix2.2.2 (K_222_) in the reactor. Subsequently, the aqueous K(K_222_)[^18^F]F solution was carefully evaporated to dryness in vacuo. An amount of ~ 5.0 mg (~ 8.4 μmol) of compound (*S*)-**9b** and carefully dried K(K_222_)[^18^F]F residue were heated at 84 °C in 1 mL DNA-grade CH_3_CN for 20 min. The mixture was cooled to 40 °C, diluted with 10 mL water for injection and passed through a Waters Sep-Pak Light C18 cartridge. The cartridge was washed with additional 10 mL water for injection and eluted with 0.5 mL warm DMF (warmed to 90 °C before elution). The eluate was diluted with 0.5 mL water for injection and purified by gradient-radio HPLC **B** using the gradient radio-HPLC method **B1**. The product fraction of compound (*S*)-[^18^F]**9a** (retention time *t*_R_ = 15.5–17.1 min) was evaporated to dryness in vacuo and redissolved with 1 mL H_2_O/EtOH (9:1) The radiosynthesis provided (*S*)-[^18^F]**9a** with an overall radiochemical yield of 31–46% (based on cyclotron-derived [^18^F]fluoride ions, *n* = 3) in 95–110 min from end of radionuclide production. (*S*)-[^18^F]**9a** was isolated in radiochemical purities of > 97% and a molar activity in the range of 2.8–3.4 GBq/μmol at the end of radiosynthesis. The molar activity was determined using analytical HPLC **C** and method **C1** (retention time of (*S*)-[^18^F]**9a**: *t*_R_ = 16.2 min).

### Determination of the partition coefficient (logD (exp.))

The lipophilicity of the radioligand (*S*)-[^**18**^**F]9a** was assessed by determination of the water/octanol partition coefficients following a published procedure (Prante et al. 2006). In brief, approximately 20 kBq of (*S*)-[^18^F]**9a** were mixed with equal amounts (0.5 mL) of PBS (pH 7.4) and 1-octanol and the resulting biphasic system was mixed vigorously for 1 min at rt. The tubes were centrifuged (3000 rpm, 2 min) and 400 μL of the octanol phase were separated and again mixed with equal amounts (400 μL) of PBS. The resulting biphasic system was again mixed vigorously of 1 min at rt. After centrifugation (3000 rpm, 2 min) three samples of 100 μL of each layer were counted in a gamma counter (Wallac Wizard, Perkin-Elmer Life Science). The partition coefficient was determined by calculating the ratio cpm(octanol)/cpm(PBS) and expressed as log*D* (exp.) (log(cpm_octanol_/cpm_PBS_)). Two independent experiments were performed in triplicate and data were provided as mean values ± standard deviation.

### In vitro enzyme inhibition assays (Table 1)

The inhibition potencies of (*R*)-**9a** and (*S*)-**9a** against activated MMP-2 and -9 were assayed using the synthetic fluorometric substrate (7-methoxycoumarin-4-yl)acetyl-Pro-Leu-Gly-Leu-(3-(2,4-dinitrophenyl)-L-2,3-diaminopropionyl)Ala-Arg-NH_2_ (R&D Systems) as described previously (Huang et al. 1997). Briefly, MMP-2 and -9 (each at 2 nM) and test compounds at varying concentrations (10 pM to 1 mM) in Tris (50 mM), pH 7.5, containing NaCl (0.2 M), CaCl_2_ (5 mM), ZnSO_4_ (20 μM), and 0.05% Brij 35 were preincubated at 37 °C for 30 min. An aliquot of substrate (10 μL of a 50 μM solution) was added to the enzyme inhibitor mixture (90 μL), and the fluorescence changes were monitored using a TriStar2 Multimode Reader LB 942, (Berthold) with excitation and emission wavelengths of 340 and 405 nm, respectively. Reaction rates were measured from the initial 10 min and plotted as a function of inhibitor concentration. From the resulting inhibition curves, the IC_50_ values were calculated by nonlinear regression analysis using the Grace 5.1.8 software (Linux).

### Biostability and metabolism study

Approximately 11.1 MBq (*S*)-[^18^F]**9a** (in a maximum volume of 200 μL) was injected into three mice each via tail vein injection. The animals were sacrificed at 30 min p.i.. Whole blood was obtained, weighed, and centrifuged at 3000 rpm (3 min) to isolate plasma. Urine was also collected. The muscle, brain, kidneys, and liver were harvested and homogenized in lysis buffer (1% SDS in PBS buffer). An aliquot of each sample (400 μL) was subsequently removed, mixed with 400 μL of acetonitrile and 100 μL of 3% acetic acid in acetonitrile, vigorously mixed, and placed on dry ice for 3 min. After thawing, the samples were centrifuged at 13000 rpm (8 min) to allow for the separation of supernatant from the pellet. The supernatant was then removed and assayed for radioactivity in a PerkinElmer Wizard γ-counter (20 s). The samples were analyzed by HPLC, using a γ-detector (Raytest GmbH/Agilent). HPLC was done on a Phenomenex C18 column (250 mm × 4.6 mm) using a gradient method with acetonitrile and water (both having 0.05% TFA).

### Animals

Adult C57/BL6 mice (male, 21–23 g) were anesthetized by isoflurane/O_2_, and one lateral tail vein was cannulated using a 27 Ga needle connected to 15 cm polyethylene catheter tubing. (*S*)-[^18^F]**9a** (500 kBq/g bodyweight) was injected as a bolus (50 μL of compound flushed with 100 μL of saline) via the tail vein, and subsequent PET scanning was performed.

### Small animal PET scanning

PET experiments were carried out using a sub-millimeter high resolution (0.7 mm full width at half maximum) small animal scanner (32 module quadHIDAC, Oxford Positron Systems Ltd., Oxford, UK) with uniform spatial resolution (< 1 mm) over a large cylindrical field (165 mm diameter, 280 mm axial length) (Schäfers et al. 2005).

List-mode data were acquired for 120 min and reconstructed into dynamic time frames using an iterative reconstruction algorithm. Subsequently, the scanning bed was transferred to the computed tomography (CT) scanner (Inveon, Siemens Medical Solutions, U.S.) and a CT acquisition with a spatial resolution of 80 μm was performed for each mouse. Reconstructed image data sets were co-registered based on extrinsic markers attached to the multimodal scanning bed and the image analysis software (Inveon Research Workplace 3.0, Siemens Medical Solutions, USA). Three-dimensional volumes of interest (VOIs) were defined over the respective organs in CT data sets, transferred to the co-registered PET data and analyzed quantitatively. Regional uptake was calculated as percentage of injected dose by dividing counts per milliliter in the VOI by total counts in the mouse multiplied by 100 (%ID/mL). Routes of tracer elimination were assessed by VOI segmentation of kidneys and bladder (renal route), and liver, gall bladder, small and large intestine (hepatobiliary route). Total activity for each route was calculated as percentage of injected dose by dividing the total counts in the respective VOI by total counts in the mouse multiplied by 100 (%ID).

## Results and discussion

Previous attempts to radiofluorinate analogs of CGS 27030A (see Fig. 1) resulted in low yields and inadmissible long synthesis times. Neither direct radiofluorination of the 3-pyridylmethyl aromatic core (Wagner et al. 2009) nor fluoroalkylation of the phenol HO-CGS 27030A in either a two- or three-step procedure using 2-[^18^F]fluoro-1-tosyloxyethane led to sufficient radiochemical yields in appropriate radiosynthesis times. In the first example, high reaction temperatures led to decomposition of the molecule and in the second example unavoidable protection and subsequent deprotection of the hydroxamic acid function resulted in prolonged radiosynthesis times (Breyholz et al. 2007). In contrast, the radiosynthesis of an analog of the CGS 25966 derivative (see Table 1) was accomplished in a one-step procedure via direct nucleophilic substitution of its tosylate precursor in good radiochemical yields (RCY: 45.6 ± 5.6%) and radiosynthesis times of 110 ± 10 min from the end of radionuclide production (Wagner et al. 2007). Furthermore, a GMP compliant fully automated radiosynthesis of the CGS 25966 derivative (RCY = 14.9 ± 3.9% in 57 ± 2 min) was prepared to enable first-in-man studies (Wagner et al. 2011).

CGS derivatives (*R*)-CGS 25966 and (*R*)-CGS 27030A show similar inhibition potencies towards MMP-2 and -9 and a substitution of the picolyl moiety by a benzyl ring does not crucially influence the binding affinities of the lead structures (Fig. 1). Both, the dissatisfying radiochemical yields for ^18^F-labelled CGS 27030A derivatives and the similar inhibition potencies of the picolyl and benzyl substituted CGS derivatives encouraged us to (radio)synthesize a benzyl variant of our γ-fluorinated α-sulfonylaminohydroxamic acid derivatives **1** and **2** (Fig. 1).

From the radiochemical point of view, introduction of [^18^F]fluorine into the γ-position of the α-amino acid is difficult. Additionally, all attempts to replace leaving groups such as tosyl or mesyl by fluoride to prepare ^18^F-labelled compound **1**, failed due to an obvious cyclization reaction (Behrends et al. 2015). Therefore, a second fluorine atom has to be introduced into the core structure analog to the radiolabeling procedure of the CGS derivative [^18^F]FEtO-CGS 25966 bearing a 2-fluoroethoxy instead of the methoxy group in *p*-position of the benzene sulfonamide group. This can be realized in a one-step procedure starting from the tosylate precursor (Wagner et al. 2011). Previous studies have shown that the *p*-fluoroethoxy motif at the benzene sulfonamide group is well tolerated without affecting the MMP inhibition potency (Hugenberg et al. 2013).

To evaluate the MMP potency of the benzyl substituted γ-fluorinated α-sulfonylaminohydroxamic acid derivative, both enantiomeric forms of the nonradioactive fluorinated target compound were synthesized and tested by in vitro fluorometric MMP assays.

### Chemistry

**Scheme 1:**
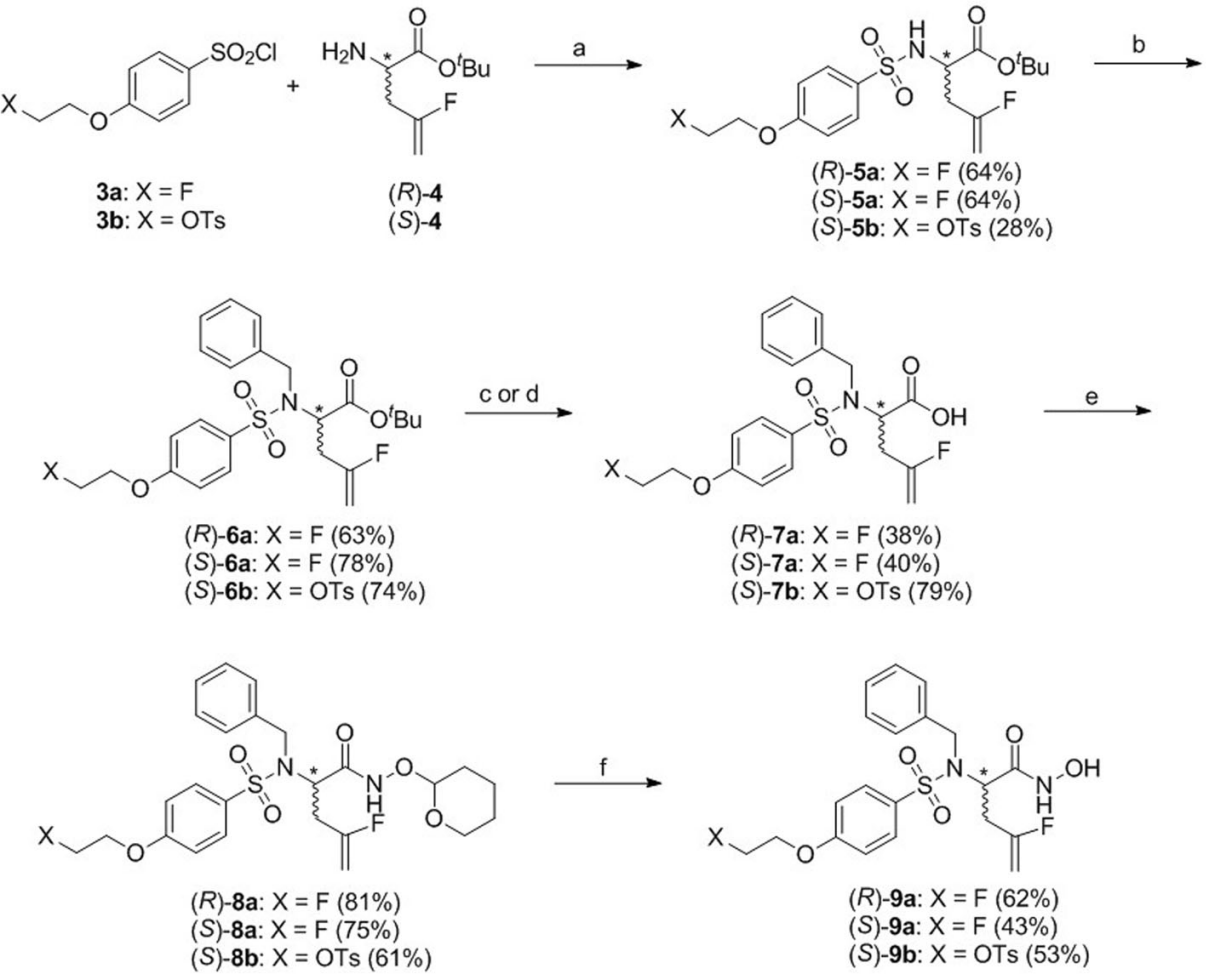
Syntheses of the MMPIs and precursor **9**: Reaction conditions: (**a**) pyridine 0 °C - rt.; (**b**) BnBr, K_2_CO_3_, DMF, rt.; (**c**) for X = F: CF_3_CO_2_H, CH_2_Cl_2_, rt.; (**d**) for X = OTs: KSF clay, CH_3_CN, reflux; (**e**) *O*-THP hydroxylamine, EDC, NMM, HOBT, DMF, rt.; (**f**) 4 N HCl in dioxane/MeOH 1:1, rt.

The syntheses of the γ-fluorinated α-sulfonylaminohydroxamic acid derivatives and the precursor for radiolabeling are depicted in Scheme 1. Building blocks for the synthesis of the MMPIs are represented by the *p*-fluoroethoxy and *p*-tosylethoxyphenylsulfonyl chlorides **3a** and **3b** as well as by the enantiomerically enriched γ-fluorinated amino acid *tert*-butyl esters (*R*)-**4** and (*S*)-**4**. *p*-(2-Fluoroethoxy)phenylsulfonyl chloride (**3a**) and 2-[4-(chlorosulfonyl)phenoxy]ethyl-4-methylbenzenesulfonate (**3b**) were prepared according to literature procedure (Wagner et al. 2011). γ-Fluorinated α-amino acid *tert*-butyl esters (*R*)-**4** and (*S*)-**4** were synthesized by diastereoselective alkylation of *Schiff’s* bases, derived from (+)-(*R*,*R*,*R*)-2-hydroxy-3-pinanone and glycine *tert*-butyl ester with 3-bromo-2-fluoropropene following a literature procedure (Laue et al. 2000). Substitution at their *N*-terminal position yielded the sulfonamides **5**. *N*-Alkylation with benzyl bromide gave carboxylic acid esters **6**. Deprotection of the fluoroethoxy substituted carboxylic acid esters (*R*)-**6a** and (*S*)-**6a** were realized using trifluoroacetic acid in dichloromethane at room temperature. Compound **6b** was deprotected differently under milder conditions using Montmorillonite KSF clay (Yadav et al. 2002) in acetonitrile under reflux. Conversion of **7** into the corresponding hydroxamic acid esters **8** were achieved by *O*-THP hydroxylamine, EDC, HOBT, and NMM in DMF. Cleavage of the THP protecting group was performed with hydrochloric acid in a dioxane/methanol mixture, yielding the γ-fluorinated hydroxamic acids **9** (Hugenberg et al. 2012).

### In vitro assays

**Fig. 2:**
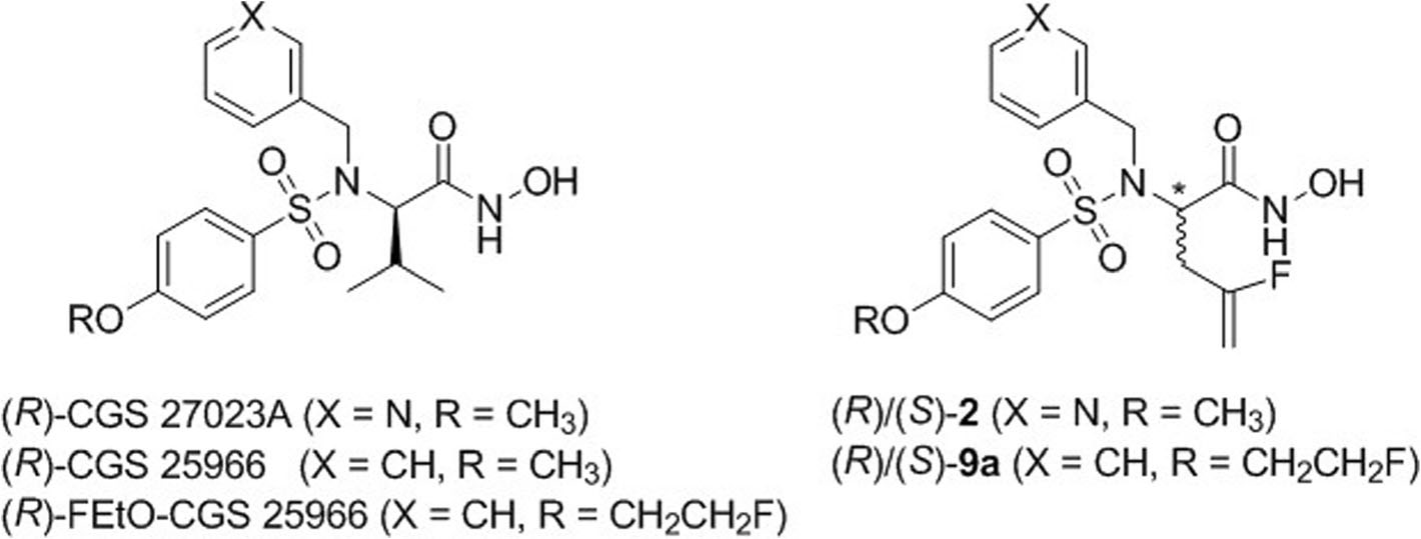
CGS lead structures and γ-fluorinated α-sulfonylaminohydroxamic acids based MMP inhibitors **2** and **9a**

The MMP inhibition potencies of the hydroxamic acids (*R*)-**9a** and (*S*)-**9a** (Fig. 2) against MMP-2 and -9 were measured by fluorometric in vitro inhibition assays, following the previously described procedure (Huang et al. 1997). The resulting IC_50_ values of the γ-fluorinated hydroxamic acids are displayed in Table 2 and were compared to those of the parent compounds (*R*)-**1** and (*S*)-**1** and the CGS lead compounds. With IC_50_ values of 10.4 nM (MMP-2) and 0.5 nM (MMP-9) for (*R*)-**9a** and 0.3 nM (MMP-2) and 0.1 nM (MMP-9) for (*S*)-**9a** the investigated MMP inhibitors show even higher inhibition potencies against MMP-2 and -9 compared to (*R*)-**1** and (*S*)-**1**. In contrast to the CGS lead compounds, where the (*R*)-enantiomers are significantly more active, (*S*)-**9a** is more potent than its (*R*)-enantiomer, which was also observed for the γ-fluorinated α-sulfonylaminohydroxamic acid (*S*)-**1**.

**Table 2:** MMP inhibition potencies and clog*D* (log*D*) values of lead structures and target novel hydroxamic acids

	IC_50_ [nM]^a^				log *D* values		
Compound	R	X	MMP-2	MMP-9	clog*D*^b^	log*D* (exp.)	Ref.
(*R*)-**CGS 27023A**	CH_3_	N	20^c^	8^c^	1.49		(Mac Pherson et al., 1997)
(*R*)-**CGS 25966**	CH_3_	CH	11^c^	27^c^	2.72		(Scozzafava and Supuran, 2000)
(*R*)-**FEtO- CGS 25966**	CH_2_CH_2_F	CH	4 ± 3	50 ± 27	2.92	2.02 ± 0.03	(Wagner et al., 2011)
(*R*)-**2**	CH_3_	N	9.3 ± 2.0	8.3 ± 0.1	0.81		(Behrends et al., 2015)
(*S*)-**2**	CH_3_	N	7.2 ± 0.1	4.9 ± 0.4	0.81		(Behrends et al., 2015)
(*R*)-**9a**	CH_2_CH_2_F	CH	10.4 ± 5.9	0.5 ± 0.3	2.23		
(*S*)-**9a**	CH_2_CH_2_F	CH	0.3 ± 0.03	0.1 ± 0.01	2.23	1.16 ± 0.2^d^	

^a^Standard deviations based on three independent experiments are given for IC_50_ values in nM range

^b^clog*D* values were calculated by ChemAxon LogD predictor, (log*D* = log*P* at physiological pH 7.4)

^c^K_i_ values, where SDs are not denoted

^d^log*D* value was determined for compound (*S*)-[^18^F]**9a**

Obviously, the substitution of the picolyl moiety by a benzyl ring and the replacement of the methoxy group in *p*-position of the benzene sulfonyl unit by a fluoroethyl group did not diminish the inhibition potencies.

Table 2 also displays the calculated log*D* values (clog*D*) of the modified γ-fluorinated hydroxamic acids to indicate the changes of the lipophilicities caused by the structural modifications. Compared to FEtO-CGS25966 (clog*D* = 2.92), the γ-fluorinated hydroxamic acids are slightly more hydrophilic. However, in comparison to the lead structure CGS27023A the calculated log*D* values indicate a shift towards higher lipophilicity. Additionally, the log*D* value of the radiofluorinated analogue (*S*)-[^18^F]**9a** (log*D*((*S*)-**9a**) = 1.16) was experimentally determined. Likewise to the lead structure [^18^F]FEtO-CGS25966 the log*D*(exp) of (*S*)-[^18^F]**9a** differs from the calculated log*D* (clog*D*((*S*)-**9a**) = 2.23) by around 1 unit. In comparison to [^18^F]FEtO-CGS25966 the γ-fluorinated hydroxamic acid (*S*)-[^18^F]**9a** is 7 times more hydrophilic, which might be caused by the fluoroallyl group.

The investigated MMP inhibitors (*R*)- and (*S*)-**9a** showed excellent MMP inhibition potencies in the nano- and subnanomolar range and similar lipophilicities similar to the lead compounds. Despite the modifications at the sulfonamide and the substitution of the picolyl unit for benzyl, the γ-fluorinated hydroxamic acid (*S*)-enantiomer is as potent as the (*R*)-enantiomer.

### Radiochemistry

**Scheme 2:**
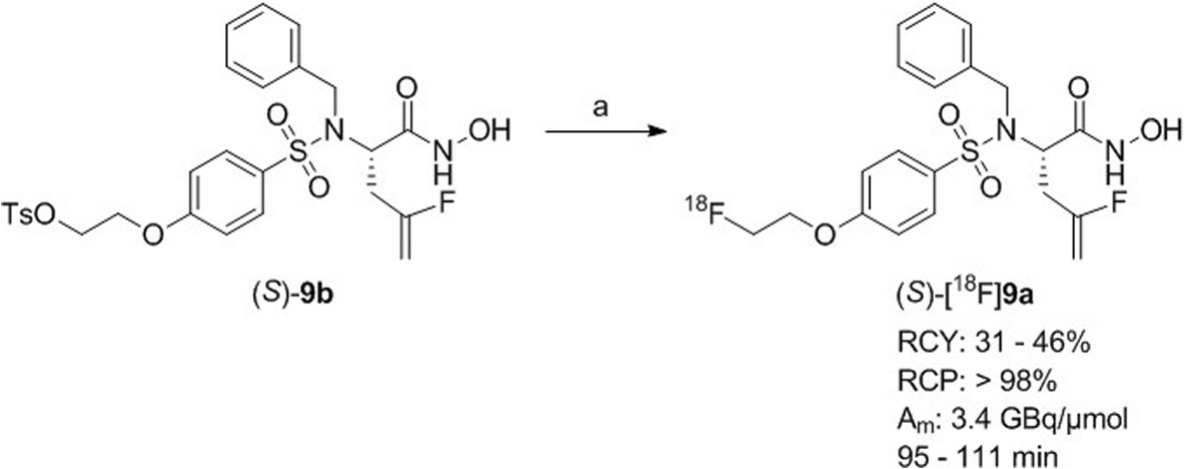
Radiosynthesis of ^18^F-labeled (*S*)-[^18^F]**9a**: Reaction conditions: (**a**) K(K_222_)[^18^F]F, K_2_CO_3_, CH_3_CN, 84 °C for 10 min

The results mentioned above encouraged us to radiosynthesize the first ^18^F-labelled (*S*)-configured γ-fluorinated MMP inhibitor based on α-sulfonylaminohydroxamic acid to further evaluate its pharmacokinetic behavior, metabolic stability and its applicability as an in vivo MMP radiotracer (Scheme 2).

Radiosynthesis of the ^18^F-labelled counterpart (*S*)-[^18^F]**9a** was accomplished via direct nucleophilic substitution reaction of the tosylate precursor (*S*)-**9b** by [^18^F]fluoride according to the synthesis described for [^18^F]FEtO-CGS 25966 (Wagner et al. 2011). The radiosynthesis provided (*S*)-[^18^F]**9a** with radiochemical yields of 31–46% (*n* = 3), radiochemical purities of > 97% in 95–110 min from the end of radionuclide production and molar activities of 2.8–3.4 GBq/μmol after radiosynthesis. The experimental log*D* value of the radioligand was determined in phosphate-buffered saline (PBS) as already described (Prante et al. 2006).

### In vivo biodistribution and metabolism study

**Fig. 3:**
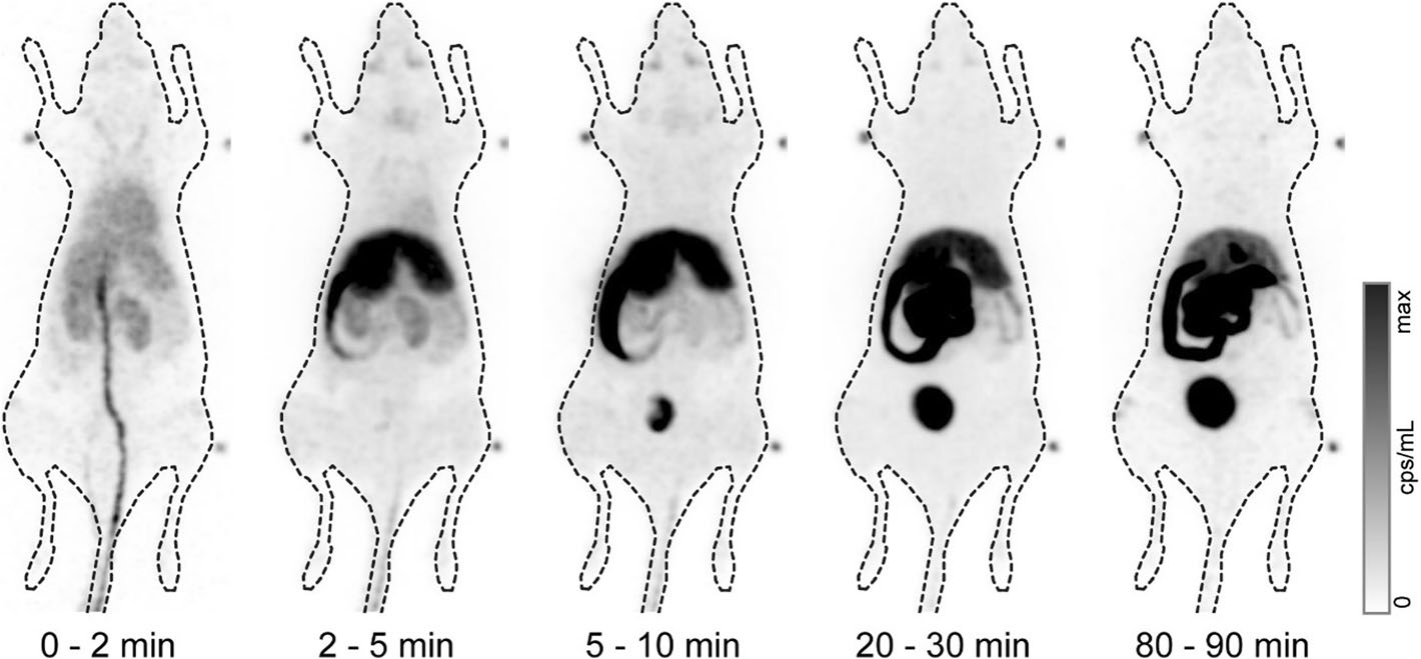
In vivo biodistribution of tracer-associated radioactivity after intravenous injection of (*S*)-[^18^F]**9a**

Maximum intensity projections (MIPs) of the whole body in vivo biodistribution of tracer-associated radioactivity in an adult C57/Bl6 mouse 0–2, 2–5, 5–10, 20–30 and 80–90 min after intravenous injection of (*S*)-[^18^F]**9a** are shown in Fig. 3.

**Fig. 4:**
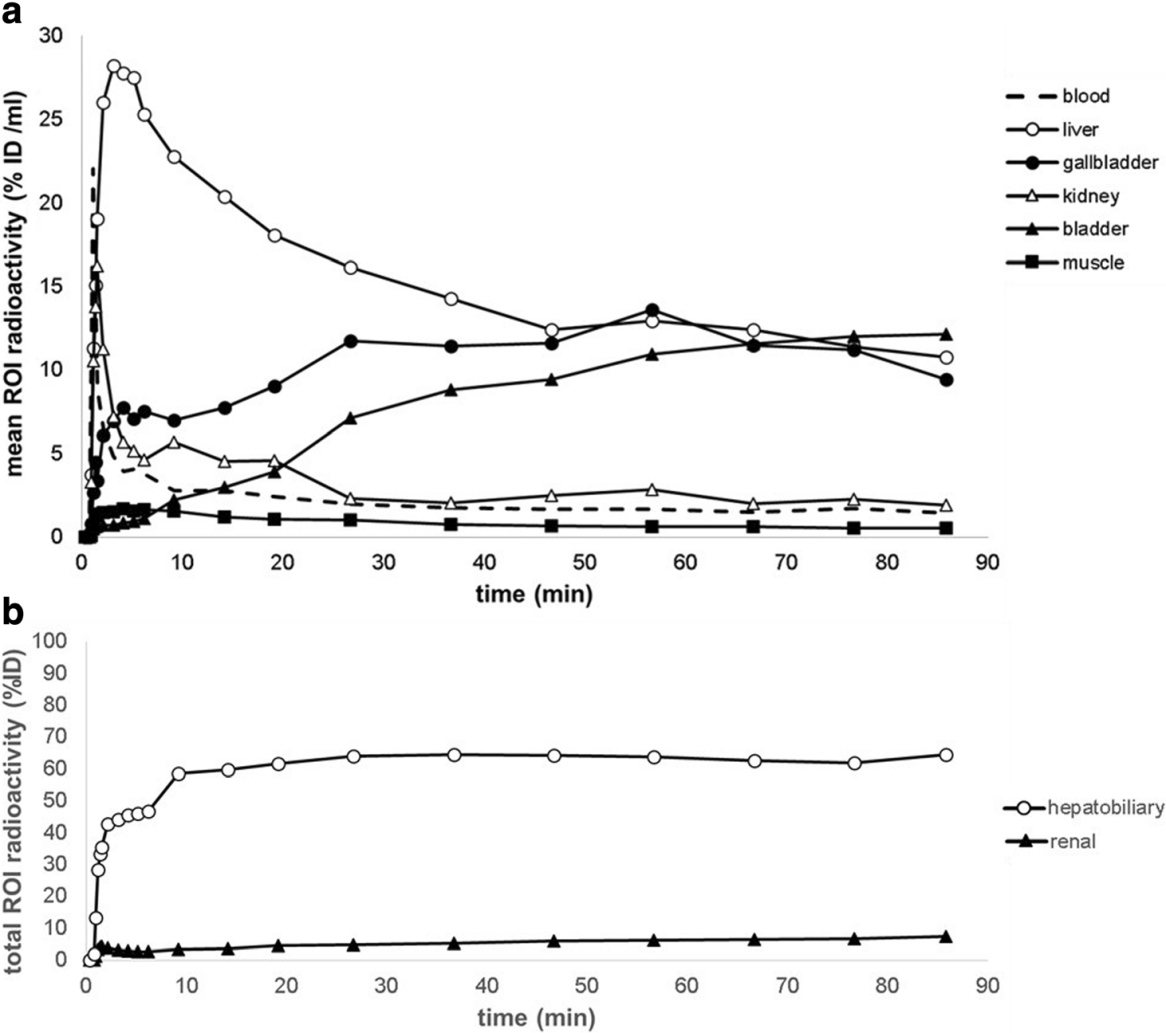
In vivo biodistribution of radioactivity in an adult C57/Bl6 mouse after intravenous injection of (*S*)-[^18^F]**9a**. **a**) Time-activity concentration curves illustrate tracer dynamics in selected regions of interests (ROI). **b**) Time-activity curves of tissue compartments involved in tracer elimination (liver, gall bladder, intestine, kidneys, bladder) demonstrate predominant hepatobiliary clearance of (S)-[^18^F]**9a** from the mouse body. % ID/mL: percentage injected dose per milliliter

Overall, (*S*)-[^18^F]**9a** is cleared fast and efficiently from the body through hepatic and renal elimination with no significant tracer remaining in non-excretion organs 80–90 min p.i (Fig. 4). Immediately upon injection of (*S*)-[^18^F]**9a** high levels of radioactivity were observed in the liver and the kidneys. While the activity in the kidney decreased (T_max_: 100 s, T_1/2_: 3 min p.i.) in parallel to the activity in the blood, the liver first showed a further accumulation of (*S*)-[^18^F]**9a** associated activity (T_max_: 3 min, T_1/2_: 36 min p.i.) before clearance into the gallbladder and finally into the intestine (Fig. 4), representing the predominant hepatobiliary route of (*S*)-[^18^F]**9a** elimination. Time-activity concentration curves of the intestinal transport of hepatobiliary eleminated (*S*)-[^18^F]**9a** is illustrated in Fig. S16 in the supporting information.

Defluorination of the radioligand in vivo potentially impairing image interpretation (indicated by bone uptake of [^18^F]fluoride ions) was not observed in the entire dynamic imaging study. Furthermore, accumulation of (*S*)-[^18^F]**9a** in organs/tissues such as the brain, myocardium, lung and muscles, indicating unspecific binding, was not observed.

**Fig. 5:**
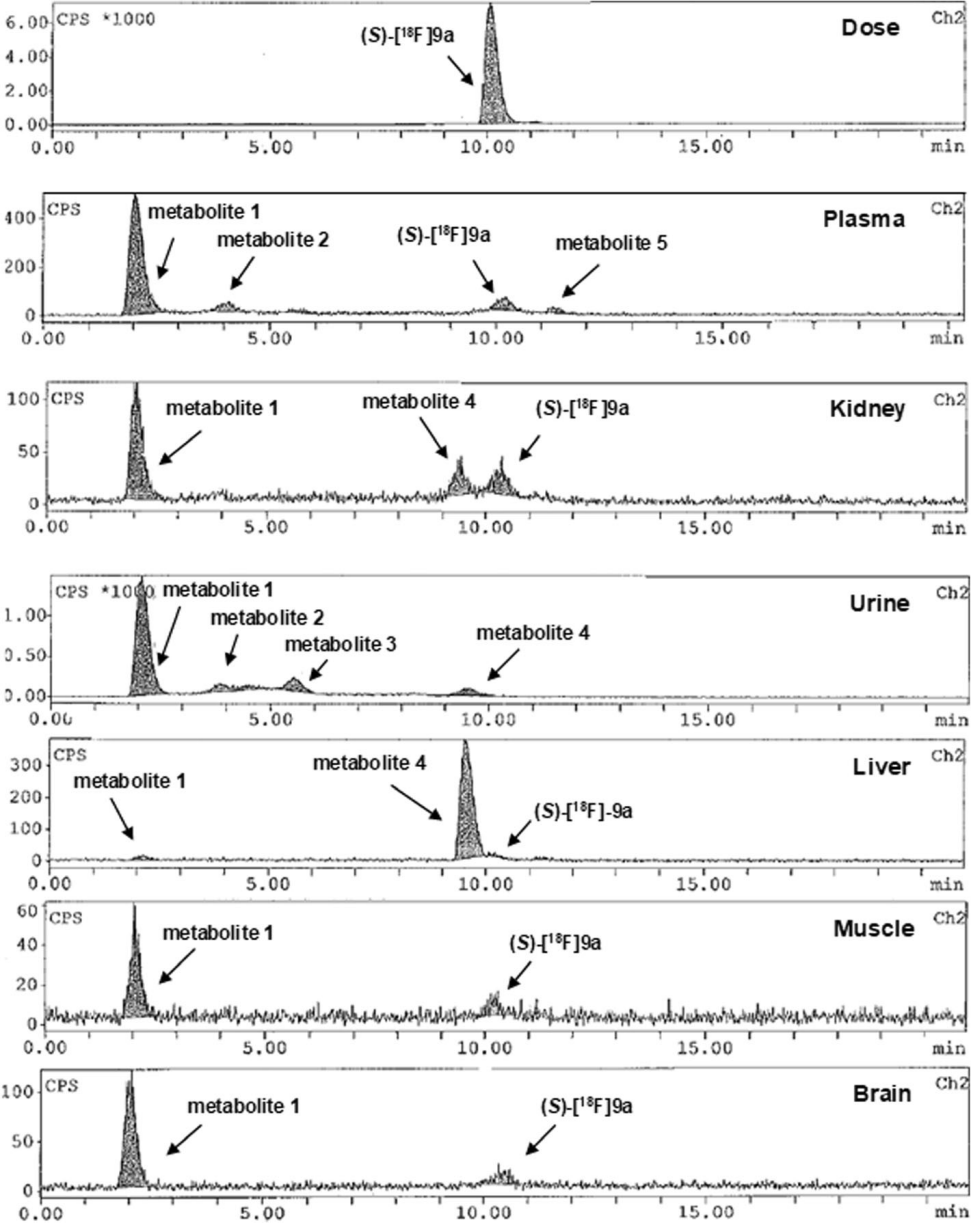
Representative radio-HPLC traces for 30 min p.i. of the metabolism study of (*S*)-[^18^F]**9a**. The radiochemical purity of (*S*)-[^18^F]**9a** was > 97% before injection. The samples were analyzed by HPLC, using a γ-detector (Raytest Isotopenmessgeräte GmbH/Agilent). The HPLC was done on a Phenomenex C18 column (250 × 4.6 mm) using a gradient method with acetonitrile and water (both having 0.05% TFA)

An in vivo biostability and metabolism study was performed in three 10-month old female OCR (CD1) wild type mice 30 min p.i. Representative radio-HPLC traces are shown in Fig. 5. The retention time of unchanged (*S*)-[^18^F]**9a** tracer was between 9.8 and 10.8 min. A total of five metabolites were detected, with retention times of 2.0 min (metabolite 1), 3.9 min (metabolite 2), 5.5 min (metabolite 3), 9.5 min (metabolite 4) and 11.4 min (metabolite 5) (Table 3).

**Table 3:** Metabolites of (*S*)-[^18^F]**9a** in plasma, kidney, urine, liver, muscle and brain

30 min p.i.	Organ/tissue/body fluid (% HPLC)					
	Plasma	Kidney	Urine	Liver	Muscle	Brain
(*S*)-[^18^F]**9a**: *t*_R_ ≈ 10.3 min	8	17	–	1	17	12
metabolite 1: *t*_R_ ≈ 2.0 min	81	66	76	4	83	88
metabolite 2: *t*_R_ ≈ 3.9 min	6	–	9		–	–
metabolite 3: *t*_R_ ≈ 5.5 min	2	–	9		–	–
metabolite 4: *t*_R_ ≈ 9.5 min	–	17	6	94	–	–
metabolite 5: *t*_R_ ≈ 11.4 min	3	–	–	1	–	–

In plasma, (*S*)-[^18^F]**9a** metabolizes to about 92% into four metabolites, three of these metabolites are more polar. Only 8% of the parent compound could be observed in plasma after 30 min in vivo in mice. In kidney homogenate, the parent tracer was present in higher amounts, i.e. 17% compared to the other tissues along with two polar metabolites. In urine, a complete metabolism of the parent compound to four polar metabolites was observed. Similar metabolism patterns were determined for muscle and brain (17% and 12% of (*S*)-[^18^F]**9a**) with only one polar metabolite formed in high amounts (83% in muscle and 88% in brain). In all samples, except the liver, the polar metabolite 1 was formed as the main compound. In contrast, in the liver (*S*)-[^18^F]**9a** showed nearly complete degradation to metabolite 4 (94%) and only 1% of the parent compound could be detected.

Recently our group published a study about radiolabeled hydroxamate-based matrix metalloproteinase inhibitors and how chemical modifications affect their pharmacokinetics and their metabolic stability. In course of this study additionally to the in vivo metabolism study an in vitro metabolism study with mouse liver microsomes was performed. (Hugenberg et al. 2016). Polar compounds like carboxylic acids (phase I transformation) and glucuronic acid derivatives (phase II) have been identified to be the main metabolites of the modified hydroxamates during this investigation. Furthermore, analogues of CGS compounds bearing a polyethylene glycol underwent decomposition by cleavage of the ethylene glycol subunits, which was reflected by very polar metabolites. Therefore we assume that our present most polar main metabolite 1, might be [^18^F]fluoroethanol or [^18^F]fluoracetic acid. Such small molecules are able to easily enter the brain (Peana et al. 2016), what furthermore can be confirmed by the fact that metabolite 1 is found to be the main metabolite in the brain fraction.

The tracer and its metabolites are predominantly cleared through the liver and the gallbladder (92%) and to a lower degree by renal excretion (8%). 90 min post injection 73% of the injected tracer activity was cleared from the blood and tissues. Compared to the lead compound CGS 25966 no significant differences in the clearance characteristics were observed.

(*S*)-[^18^F]**9a** metabolizes in plasma, kidneys, urine, muscle and brain to about 90% and the polar metabolite 1 was formed as the major metabolite. In contrast, in the liver a nearly complete degradation of the parent compound to the slightly more polar metabolite 4 was observed.

## Conclusion

In a six step sequence, two new enantiomerically pure MMP-2 and -9 inhibitors were synthesized in 7.7% and 6.4% overall yield, respectively. As a consequence of the presence of a fluorine atom in the amino acid core, both enantiomers are biologically almost equally active, likely attributable to additional interactions of the fluorine with the active site of the enzymes. Surprisingly, the (*S*)-enantiomer was even more active than the (*R*)-isomer in contrast to the CGS lead structures. Also, the new inhibitors are more hydrophilic (experimental log*D* = 1.2) compared to the analog derived from CGS 25966 (experimental log*D* = 2.0). The most active inhibitor was ^18^F-labelled (*S*)-[^18^F]**9a** (38% radiochemical yield, > 97% radiochemical purity, 3.4 GBq/μmol molar specific activity, 102 min synthesis time). Despite higher hydrophilicity, the in vivo biodistribution of (*S*)-[^18^F]**9a** possess no significant differences, compared to the slightly more lipophilic ^18^F-fluoroethoxy substituted lead compound CGS 25966. Unfortunately, this radiotracer is metabolically not stable in wild type mice, which impairs further clinical development of this tracer. Two major radioactive metabolites were found in different concentrations in different organs. However, metabolic defluorination was not observed. Higher metabolic stability was found for CGS-based MMPIs bearing a 1,4-disubstituted 1,2,3-triazole core instead of the benzyl moiety. Therefore, metabolic stability of (*S*)-[^18^F]**9a** could possibly be improved by the substitution of the benzyl ring by a triazole moiety.

## Additional file

Additional file 1: (Experimental procedures, analytical data for compounds (*R*)-**5a**, (*S*)-**5b**, (*R*)-**6a**, (*S*)-**6b**, (*R*)-**7a**, (*S*)-**7b**, (*R*)-**8a**, (*S*)-**8b**, (*R*)-**9a** and (*S*)-**9b**, and copies of NMR spectra) associated with this article can be found at. (DOCX 4305 kb)

## Abbreviations

% ID: percentage injected dose
CH: Cyclohexane
EA: Ethyl acetate
EDC: *N′’*-ethyl*-N′’*-(3-dimethylaminopropyl)-carbodiimide hydrochloride
EM: Exact mass
GMP: Good Manufacturing Practice
HOBT: 1-hydroxy-benzotriazole
ICR: Imprinting control region
K_222_: Kryptofix2.2.2
log *D*: log *P* at physiological pH (7.4)
MMP: Matrix metalloproteinase
MMPI: Matrix metalloproteinase inhibitor
NMM: 4-methyl-morpholine
p.i.: Post injection
PET: Positron emission tomography
RCP: Radiochemical product
RCY: Radiochemical yield (decay corrected)
ROI: Region of interest
SD: Standard deviation
SPECT: Single photon emission computed tomography
THP: Tetrahydropyranyl
TIMP: Tissue inhibitors of metalloproteinase
*t*_R_: Retention time
