# Supplementary material for: Synthesis, radiosynthesis, in vitro and first in vivo evaluation of a new matrix metalloproteinase inhibitor based on γ-fluorinated α-sulfonylaminohydroxamic acid
Source: EJNMMI Radiopharm Chem. 2018 Jul 27;3:10. doi: 10.1186/s41181-018-0045-0 (PMC6063323; doi:10.1186/s41181-018-0045-0)
Supplement: Supplementary file 1 — (Experimental procedures, analytical data for compounds (R)-5a, (S)-5b, (R)-6a, (S)-6b, (R)-7a, (S)-7b, (R)-8a, (S)-8b, (R)-9a and (S)-9b, and copies of NMR spectra) associated with this article can be found at. (DOCX 4305 kb) [file 41181_2018_45_MOESM1_ESM.docx]

**Additional file 1**

**Synthesis, radiosynthesis, *in vitro* and first *in vivo* evaluation of a new matrix metalloproteinase inhibitor based on γ-fluorinated α-sulfonylaminohydroxamic acid**

Verena Hugenberg^a,b,†*^, Malte Behrends^c^, Stefan Wagner^b^, Sven Hermann^a,d^, Michael Schäfers^a,b,d^, Hartmuth C. Kolb^e^, Katrin Szardenings^e^, Joseph C. Walsh^e^, Luis F. Gomez^e^, Klaus Kopka^b,††^, Günter Haufe^a,c,d*^

^a^ European Institute for Molecular Imaging, University of Münster, Waldeyerstr. 15, D-48149 Münster, Germany

^b^ Department of Nuclear Medicine, University Hospital Münster, Albert-Schweitzer-Campus 1, Building A1, D-48149 Münster, Germany

^c^ Organisch-Chemisches Institut, Westfälische Wilhelms-Universität Münster, Corrensstraße 40, D-48149 Münster, Germany

^d^ ‘Cells in Motion’ Cluster of Excellence, University of Münster, Waldeyerstr. 15, D-48149 Münster, Germany

^e^ Siemens Medical Solutions USA, Inc., 6140 Bristol Parkway, Culver City, California 90230, USA.

^†^Current address: Institute for Radiology, Nuclear Medicine and Molecular Imaging, Heart and Diabetes Center North Rhine Westphalia, University Hospital, Ruhr University Bochum, Georgstraße 11, D-32545 Bad Oeynhausen, Germany

^††^Current address: German Cancer Research Center (dkfz), Division of Radiopharmaceutical Chemistry, Im Neuenheimer Feld 280, D-69120 Heidelberg, Germany.

*1. Experimental section*

*1.1. General methods and chemistry*

All chemicals, reagents and solvents for the synthesis of the compounds were analytical grade, purchased from commercial sources and used without further purification unless otherwise specified. All air and moisture-sensitive reactions were performed under argon atmosphere. Solvents were purified and dried analog to literature methods, where necessary. The melting points (mp) are uncorrected and were determined in capillary tubes on a Stuart Scientific SMP3 capillary melting point apparatus. Column chromatography was performed on Merck silica gel 60 (0.040 – 0.063 mm). Thin layer chromatography (TLC) was carried out on silica gel-coated polyester backed TLC plates (Polygram, SIL G/UV_254_, Macherey-Nagel) using solvent mixtures of cyclohexane (CH), ethyl acetate (EA) and methanol (MeOH). Compounds were visualized by UV light (254 nm). NMR spectra were recorded in CDCl_3_, CD_3_OH or DMSO-*d_6_* on a Bruker ARX300, a Bruker DPX300 (^1^H NMR, 300 MHz, ^13^C NMR, 75MHz, ^19^F NMR, 282 MHz), a Bruker AMX 400 (^1^H NMR, 400 MHz, ^13^C NMR, 100 MHz) and a Varian Unity plus 600 (^1^H NMR, 600 MHz, ^13^C NMR, 151 MHz) spectrometer. TMS (^1^H), CDCl_3_, DMSO-*d_6_*, CD_3_OD (^13^C) and CFCl_3_ (^19^F) were used as internal standards and all chemical shift values were recorded in ppm (*δ*). Exact mass analyses were conducted on a Bruker MicroTof apparatus. The chemical and radiochemical purities of each new non-radioactive and radioactive compound were ≥ 95% and assessed by analytical gradient reversed-phase HPLC system **A**. HPLC system **A**: Two K-1800 pumps and an S-2500 UV detector (Herbert Knauer GmbH), a GabiStar γ-detector (Raytest Isotopenmessgeräte GmbH). The recorded data were processed by the ChromGate HPLC software (Herbert Knauer GmbH). The HPLC method **A1** started with a linear gradient from 10% to 90% CH_3_CN in water (0.1% TFA) over 9 min, followed by a linear gradient from 90% to 10% CH_3_CN in water (0.1% TFA) over 6 min, with a flow rate of 1 mL·min^-1^.

*p*-(2-Fluoroethoxy)phenylsulfonyl chloride (**3a**) (Wagner et al. 2011), 2-[4-(chlorosulfonyl)phenoxy]ethyl-4-methylbenzenesulfonate (**3b**) (Wagner et al. 2011), *tert*-butyl (*S*)-2-amino-4-fluoropent-4-enecarboxylate ((*S*)-**4**) (Laue et al. 2000) and *tert*-butyl (*R*)-2-amino-4-fluoropent-4-enecarboxylate ((*S*)-**4**) (Laue et al. 2000) were synthesized following literature procedures. All animal experiments were conducted in accordance with local institutional guidelines for the care and use of laboratory animals.

*2. Preparation of MMPI (R)-****9a***

*2.1. tert-Butyl (R)-4-fluoro-2-{[4-(2-fluoroethoxy)phenyl]sulfonamide}pent-4-enoate ((R)-****5a****)*. Prepared from *tert*-butyl (*R*)-2-amino-4-fluoropent-4-enecarboxylate (1000 mg, 5.28 mmol) and *p*-(2-fluorethoxy)phenylsulfonyl chloride (1225 mg, 5.28 mmol). Yield: 1.32 g (3.37 mmol, 64 %); mp 102 °C (EtOAc/Cy). ^1^H NMR (300 MHz, CDCl_3_): δ= 7.79 (dm, ^3^*J*_H,H_ = 9.0 Hz, Ar*H,* 2H), 6.99 (dm, ^3^*J*_H,H_ = 9.0 Hz, Ar*H,* 2H), 5.37 (d, ^3^*J*_H,H_ = 8.9 Hz, N*H*, 1H), 4.78 (dm, ^2^*J*_H,F_ = 47.3 Hz, C*H*_2_F, 2H), 4.64 (dd, ^3^*J*_H,F_ = 17.0 Hz, ^2^*J*_H,H_ = 3.1 HzCF=C*H*_2_, H*_cis_*, 1H,), 4.35 (dd, ^3^*J*_H,F_ = 49.3 Hz, ^2^*J*_H,H_ = 3.1 HzCF=C*H*_2_, *H_trans_*, 1H,), 4.26 (dm, ^3^*J*_H,F_ = 27.7 Hz, CH_2_FC*H*_2_, 2H), 3.98 (dt, ^3^*J*_H,H_ = 9.0 Hz, ^3^*J*_H,H_ = 5.9 Hz, NHC*H*, 1H), 2.65 (d, ^3^*J*_H,H_ = 5.9 Hz, NHCHC*H*_2_, H_A_, 1H), 2.59 (dd, ^3^*J*_H,H_ = 5.7 Hz, ^3^*J*_H,H_ = 4.2 Hz, NHCHC*H*_2_, H_B_, 1H), 1.30 (sC(C*H*_3_)_3_, 9H,). ^13^C NMR (75 MHz, CDCl_3_): δ = 169.2 (*C*O), 161.6 (Ar*C*O), 160.9 (d, ^1^*J*_C,F_ = 257.1 Hz, *C*H_2_F), 131.9 (Ar*C*SO_2_), 129.5 (Ar*C*H), 114.7 (Ar*C*H), 94.1 (d, ^2^*J*_C,F_ = 18.8 Hz, *C*H_2_=CF), 83.2 (*C*(CH_3_)_3_), 67.4 (d, ^2^*J*_C,F_ = 20.4 Hz, *C*H_2_CH_2_F), 53.2 (NH*C*H), 36.4 (d, ^2^*J*_C,F_ = 27.4 Hz, NHCH*C*H_2_), 27.6 (C(*C*H_3_)_3_). ^19^F NMR (282 MHz, CDCl_3_): δ = -96.1 (ddt, ^3^*J*_H,F_ = 49.3 Hz, ^3^*J*_H,F_ = 20.4 Hz, ^3^*J*_H,F_ = 17.2 Hz, CH_2_F), -224.3 (tt, ^2^*J*_H,F_ = 47.3 Hz, ^3^*J*_H,F_ = 27.8 Hz, CH_2_F). Elemental analysis: C_17_H_23_F_2_NO_5_S (M = 391.43 g/mol), calcd. C 52.16, H 5.92, N 3.58; found C 52.64, H 6.24, N 3.55 %. MS-ES(+)-EM: *m/z =* 414.1160 [(M+Na)^+^] calcd. for C_17_H_23_F_2_NO_5_SNa^+^: 414.1163.

*2.2. tert-Butyl (R)-2-{[N-benzyl-4-(2-fluoroethoxy)phenyl]sulfonamide}-4-fluoropent-4-enoate ((R)-****6a****)* Prepared from *tert*-butyl *N*-[*p*-(2-fluoroethoxy)phenylsulfonyl]aminopent-4-enoate ((*R*)-**5a**, 783 mg, 2 mmol), benzyl bromide (342 mg, 2 mmol) and potassium carbonate (2.8 g, 20 mmol). Yield: 600 mg (1.2 mmol, 63 %). ^1^H NMR (300 MHz, CDCl_3_): δ = 7.82 (dm, ^3^*J*_H,H_ = 9.0 Hz, Ar*H*, 2H), 7.29 (m, Ar*H*, 5H), 6.98 (dm, ^3^*J*_H,H_ = 9.0 Hz, Ar*H*, 2H), 4.80 (dm, ^2^*J*_H,F_ = 47.1 Hz, CH_2_F, 2H), 4.66 (d, ^2^*J*_H,H_ = 16.3 Hz, NC*H*_2_, H_A_, 1H), 4.56 (t, ^3^*J*_H,H_ = 7.3 Hz, NC*H*, 1H), 4.53 (dd, ^3^*J*_H,F_ = 17.1 Hz, ^2^*J*_H,H_ = 2.9 Hz, CF=C*H*_2_, H*_cis_*, 1H), 4.29 (d, ^2^*J*_H,H_ = 15.9 Hz, NCHC*H*_2_, H_B_, 2H), 4.27 (dm, ^3^*J*_H,F_ = 27.8 Hz, C*H*_2_CH_2_F, 2H), 4.11 (dd, ^3^*J*_H,F_ = 49.8 Hz, ^2^*J*_H,H_ = 3.1 Hz, CF=C*H*_2_, H*_trans_*, 1H), 2.70 (dt, ^3^*J*_H,F_ = 15.4 Hz, ^3^*J*_H,H_ = 6.9 Hz, NCHC*H*_2_, H_A_, 1H), 2.46 (ddd, ^3^*J*_H,F_ = 20.0 Hz, ^2^*J*_H,H_ = 15.1 Hz, ^3^*J*_H,H_ = 7.6 Hz, NCHC*H*_2_, H_B_, 1H), 1.36 (s, C(C*H*_3_)_3_, 9H). ^13^C NMR (75 MHz, CDCl_3_): δ = 168.5 (*C*O), 161.9 (d, ^1^*J*_C,F_ = 256.5 Hz, *C*F), 161.7 (Ar*C*O), 136.7 (Ar*C*CH_2_), 132.5 (Ar*C*SO_2_), 129.9 (Ar*C*H), 128.5 (Ar*C*H), 128.4 (Ar*C*H), 127.7 (Ar*C*H), 114.6 (Ar*C*H), 93.1 (d, ^2^*J*_C,F_ = 19.1 Hz, *C*H_2_=CF), 82.6 (*C*(CH_3_)_3_), 81.5 (d, ^1^*J*_C,F_ = 171.7 Hz, *C*H_2_F), 67.4 (d, ^2^*J*_C,F_ = 20.5 Hz, *C*H_2_CH_2_F), 57.6 (N*C*H), 50.0 (N*C*H_2_), 34.1 (d, ^2^*J*_C,F_ = 27.9 Hz, NCH*C*H_2_), 27.8 (C(C*H*_3_)_3_). ^19^F NMR (282 MHz, CDCl_3_): δ = -97.7 (m, ^3^*J*_H,F_ = 49.8 Hz, ^3^*J*_H,F_ = 20.0 Hz, ^3^*J*_H,F_ = 17.0 Hz, CF), -224.3 (tt, ^2^*J*_H,F_ = 47.3 Hz, ^3^*J*_H,F_ = 27.6 Hz, CH_2_F). MS-ES(+)-EM: *m/z =* 504.1615 [(M+Na)^+^] calcd. for C_24_H_29_F_2_NO_5_SNa^+^: 504.1632.

*2.3. (R)-2-{[N-Benzyl-4-(2-fluoroethoxy)phenyl]sulfonamide}-4-fluoropent-4-enoic acid ((R)-****7a****)* Prepared from *tert*-butyl *N-*benzyl-(*R*)-*N*-[*p*-(2-fluoroethoxy)phenylsulfonyl]-2-amino-4-fluoropent-4-enoate ((*R*)-**6a**) (300 mg 0.70 mmol). Yield: 115 mg (0.27 mmol, 0.38%). ^1^H NMR (300 MHz, CD_3_CN): δ = 7.78 (dm, ^3^*J*_H,H_ = 9.0 Hz, Ar*H*, 2H), 7.30 (m, Ar*H*, 5H), 7.04 (dm, ^3^*J*_H,H_ = 9.0 Hz, Ar*H*, 2H), 4.76 (dm, ^2^*J*_H,F_ = 47.7 Hz, CH_2_F, 2H), 4.65 (dd, ^3^*J*_H,H_ = 8.5 Hz, ^3^*J*_H,H_ = 6.0 Hz, NC*H*, 1H), 4.57 (d, ^2^*J*_H,H_ = 16.1 Hz, NC*H*_2_, H_A_, 1H), 4.52 (dd, ^3^*J*_H,F_ = 17.8 Hz, ^2^*J*_H,H_ = 3.0 Hz, CF=C*H*_2_, H*_cis_*, 1H), 4.33 (d, ^2^*J*_H,H_ = 16.1 Hz, NC*H*_2_, H_B_, 1H), 4.29 (dm, ^3^*J*_H,F_ = 29.5 Hz, C*H*_2_CH_2_F, 2H), 4.14 (dd, ^3^*J*_H,F_ = 51.5 Hz, ^2^*J*_H,H_ = 3.5 Hz, CF=C*H*_2_, H*_trans_*, 1H), 2.76 (ddd, ^3^*J*_H,F_ = 14.8 Hz, ^2^*J*_H,H_ = 13.7 Hz, ^3^*J*_H,H_ = 6.0 Hz, NC*H*_2_, H_A_, 1H), 2.48 (ddd, ^3^*J*_H,F_ = 22.3 Hz, ^2^*J*_H,H_ = 15.4 Hz, ^3^*J*_H,H_ = 8.5 Hz, NHC*H*_2_, H_B_, 1H). ^13^C NMR (75 MHz, CD_3_CN): δ = 171.2 (*C*O), 163.2 (ds, ^1^*J*_C,F_ = 254.7 Hz, *C*F), 163.1 (Ar*C*O), 138.2 (Ar*C*CH_2_), 133.0 (Ar*C*SO_2_), 131.0 (Ar*C*H), 129.6 (Ar*C*H), 129.3 (Ar*C*H), 128.7 (Ar*C*H), 115.7 (Ar*C*H), 93.8 (d, ^2^*J*_C,F_ = 18.9 Hz, CF=*C*H_2_), 83.1 (d, ^1^*J*_C,F_ = 167.3 Hz, *C*H_2_F), 68.8 (d, ^2^*J*_C,F_ = 19.4 Hz, *C*H_2_CH_2_F), 57.9 (N*C*H), 50.8 (N*C*H_2_), 34.1 (d, ^2^*J*_C,F_ = 28.2 Hz, NCH*C*H_2_). ^19^F NMR (282 MHz, CD_3_CN) δ = -97.7 (dddd, ^3^*J*_H,F_ = 51.0 Hz, ^3^*J*_H,F_ = 22.1 Hz, ^3^*J*_H,F_ = 17.7 Hz, ^3^*J*_H,F_ = 13.4 Hz, CF), -224.0 (tt, ^2^*J*_H,F_ = 47.7 Hz, ^3^*J*_H,F_ = 29.5 Hz, CH_2_F). MS-ES(+)-EM: *m/z =* 448.1004 [(M+Na)^+^] calcd. for C_20_H_31_F_2_NO_5_SNa^+^: 448.1006.

*2.4. (2R)-2-[N-Benzyl-4-(2-fluoroethoxy)phenylsulfonamido]-4-fluoro-N-[(tetrahydro-2H-pyran-2-yl)oxy]pent-4-enamide ((R)-****8a****).* Prepared from (*R*)-2-{[N-Benzyl-4-(2-fluoroethoxy)phenyl]sulfonamide}-4-fluoropent-4-enoic acid ((*R*)-**7a**), 1‑hydroxybenzotriazole hydrate (HOBT, 1.2 eq., 38 mg, 0.282 mmol), 4‑methylmorpholine (NMM, 3.0 eq., 78 µL, 249.70 mmol), *O*-tetrahydro-2-*H*-pyran-2-yl-hydroxylamine (3.1 eq., 85 mg, 0.729 mmol and *N*-[(dimethylamino)-propyl]-*N*’-ethylcarbodiimide hydrochloride (EDC, 1.4 eq., 63 mg, 0.329 mmol). Yield: 101 mg (0.190 mmol, 81%). ^1^H NMR (300 MHz, CDCl_3_): δ = 9.08 (s, N*H*, 1H), 7.77 (d, ^3^*J*_H,H_ = 9.0 Hz, Ar*H*, 2H), 7.72 (d, ^3^*J*_H,H_ = 9.0 Hz, Ar*H*, 2H), 7.39 – 7.21 (m, Ar*H*, 5H), 6.98 (d, ^3^*J*_H,H_ = 9.0 Hz, Ar*H*, 2H), 6.97 (d, ^3^*J*_H,H_ = 9.0 Hz, Ar*H*, 2H), 4.92 – 4.88 (m, NHOC*H*O), 4.80 (dm, ^2^*J*_H,F_ = 47.3 Hz, C*H*_2_F, 2H), 4.66 (AB, m, NC*H*_2_, 1H), 4.58 – 4.45 (m, NC*H*, NC*H*_2,_ 2H), 4.45 – 4.34 (m, CF=C*H*_2_*,* H*_cis_*, 1H), 4.28 (dm, ^3^*J*_H,F_ = 27.7 Hz, O-C*H*_2_CH_2_F, 2H), 4.10 (dd, CF=C*H*_2_*,* H*_trans_*, ^3^*J*_H,F_ = 49.9 Hz, ^2^*J*_H,F_ = 3.0 Hz, 1H), 3.95 – 3.84 (m, NHOCHOC*H*_2_, 1H), 3.70 – 3.58 (m, NHOCHOC*H*_2_, 1H), 2.92 – 2.64 (AB, m, NCHC*H*_2_, 1H), 2.43 – 2.18 (AB, m, NCHC*H*_2_, 1H), 1.88 – 1.50 (m, THP-C*H*_2_, 6H). ^13^C NMR (75 MHz, CDCl_3_): δ = 165.8 (CONH), 165.7 (CONH), 162.0 (qAr*C*OCH_2_CH_2_F), 161.3 (d, ^1^*J*_C,F_ = 256.1 Hz, *C*FCH_2_), 136.6 (qAr*C*CH_2_N), 136.4 (qAr*C*CH_2_N), 131.6 (qAr*C*SO_2_), 129.8 (Ar*C*H), 129.8 (Ar*C*H), 128.8 (Ar*C*H), 128.7 (Ar*C*H), 128.5 (Ar*C*H), 128.5 (Ar*C*H), 127.90, (Ar*C*H), 127.86 (Ar*C*H), 114.8 (Ar*C*H), 114.8 (Ar*C*H), 102.2 (NHO*C*HO), 101.8 (NHO*C*HO), 93.8 (d, ^2^*J*_C,F_ = 18.9 Hz, CF*C*H_2_), 81.4 (^1^*J*_C,F_ = 171.9 Hz, OCH_2_*C*H_2_F), 67.4 (d, ^2^*J*_C,F_ = 20.5 Hz, O*C*H_2_CH_2_F), 62.13 (NHOCHO*C*H_2_), 62.08 (NHOCHO*C*H_2_), 54.7 (N*C*H), 54.6 (N*C*H), 48.7 (N*C*H_2_), 48.5 (N*C*H_2_), 32.2 (d, ^2^*J*_C,F_ = 27.6 Hz, NCH*C*H_2_CF), 27.7 (NHOCH*C*H_2_), 24.9 (CH_2_), 18.2 (NHOCHCH_2_*C*H_2_). ^19^F NMR (282 MHz, CDCl_3_): δ = -97.51 and 97.77 (m, CFCH_2_, 1F), -223.89 and 223.90 (tt, ^2^*J*_H,F_ = 47.3, ^3^*J*_H,F_ = 27.6 Hz, OCH_2_CH_2_F, 1F). MS-ES-EM *m/z* = 547.1695 [(M+Na)^+^] calcd for C_25_H_30_F_2_N_2_O_6_SNa^+^: 547.1685.

*2.5. (R)-2-[N-Benzyl-4-(2-fluoroethoxy)phenylsulfonamido]-4-fluoro-N-hydroxypent-4-enamide ((R)-****9a****).* The reaction was carried out in a 0.190 mmol scale with (2*R*)-2-(*N*-benzyl-4-(2-fluoroethoxy)phenylsulfonamido)-4-fluoro-*N*-((tetrahydro-2*H*-pyran-2-yl)oxy)pent-4-enamide ((*R*)-**8a**). After column chromatography a light brown wax was obtained. Yield: 52 mg (0.076 mmol, 62%). δ = 9.25 (s, 1 OH), 7.71 (d, ^3^*J*_H,H_ = 8.8 Hz, Ar*H*, 2H), 7.38 – 7.18 (m, Ar*H*, 5H), 6.96 (d, ^3^*J*_H,H_ = 8.8 Hz, Ar*H*, 2H), 4.79 (dm, ^2^*J*_H,F_ = 47.2 Hz, C*H*_2_F, 2H), 4.54 (AB, d, ^2^*J*_H,H_ = 15.5 Hz, NC*H*_2_, 1H), 4.52 (m, NC*H*, 1H), 4.46 (AB, d, ^2^*J*_H,H_ = 15.8 Hz, NC*H*_2_, 1H), 4.38 (dd, CFC*H*_2_, H*_cis_*, ^3^*J*_H,F_ = 17.0 Hz, ^2^*J*_H,F_ = 2.8 Hz, 1H), 4.26 (dm, ^3^*J*_H,F_ = 27.3 Hz, CH_2_FC*H*_2_, 2H), 4.08 (dd, CFC*H*_2_*,* H*_trans_*, ^3^*J*_H,F_ = 50.2 Hz, ^2^*J*_H,F_ = 2.2 Hz, 1H), 2.86 – 2.63 (AB, m, NCHC*H*_2_, 1H), 2.47 – 2.25 (AB, m, NCHC*H*_2_, 1H). ^13^C NMR (101 MHz, CDCl_3_): δ = 166.4 (CONH), 162.1 (qAr*C*OCH_2_CH_2_F), 161.1 (d, ^1^*J*_C,F_ = 256.3 Hz, *C*FCH_2_), 136.2 (qAr*C*CH_2_N), 131.3 (qAr*C*SO_2_), 129.7 (Ar*C*H), 128.60 (Ar*C*H), 128.6 (Ar*C*H), 128.0 (Ar*C*H), 114.8 (Ar*C*H), 93.7 (d, ^2^*J*_C,F_ = 19.4 Hz, CF*C*H_2_), 81.4 (d, ^1^*J*_C,F_ = 171.6 Hz, OCH_2_*C*H_2_F), 67.4 (d, ^2^*J*_C,F_ = 20.4 Hz, O*C*H_2_CH_2_F), 54.2 (N*C*H), 48.66 (N*C*H_2_), 31.8 (d, ^2^*J*_C,F_ = 27.8 Hz, NCH*C*H_2_CF). ^19^F NMR (282 MHz, CDCl_3_): δ = -97.54 (m, CFCH_2_, 1F), -223.83 (tt, ^2^*J*_H,F_ = 47.3, ^3^*J*_H,F_ = 27.6 Hz, OCH_2_CH_2_F, 1F). MS-ES-EM *m/z* = 463.11049 [(M+Na)^+^] calcd for C_20_H_22_F_2_N_2_O_5_SNa^+^: 463.11097. HPLC t*_R_* = 9.10 min (99%).

*3. Preparation of Precursor (S)-****9b***

*3.1. tert-Butyl (2S)-4-fluoro-2-{4-[2-(tosyloxy)ethoxy]phenylsulfonamido}pent-4-enoate ((S)-****5b****). tert*-Butyl (2*S*)-2-amino-4-fluoropent-4-enoate (2.00 g, 10.57 mmol) was dissolved in pyridine (4 mL). After cooling to 0 °C 2-[4-(chlorosulfonyl)phenoxy]ethyl-4-methylbenzenesulfonate (1.0 eq., 4.13 g, 10.57 mmol) was added to the stirred solution. The mixture was allowed to warm up to room temperature and was stirred overnight. Subsequently the mixture was diluted with dichloromethane (10 mL) and washed with aqueous 1N HCl and brine. After drying over magnesium sulfate the solvent was removed under reduced pressure. Column chromatographic purification (silica gel, cyclohexane/ethyl acetate, 6:1) gave the sulfonamide as a colorless solid. Yield:1.61 g (28%); mp 124.8 °C. ^1^H NMR (300 MHz, CDCl_3_): δ = 7.81 (d, ^3^*J*_H,H_= 8.3 Hz, Ar*H*, 2H), 7.75 (d, ^3^*J*_H,H_ = 9.0 Hz, Ar*H*, 2H), 7.36 (d, ^3^*J*_H,H_= 8.0 Hz, Ar*H*, 2H), (6.85 (d, ^3^*J*_H,H_= 9.0 Hz, Ar*H*, 2H), 5.27 (d, ^3^*J*_H,H_= 8.9 Hz, N*H*, 1H), 4.64 (dd, ^3^*J*_H,F_ = 17.1 Hz, ^2^J_H,H_ = 3.1 Hz, CFC*H*_2_,H*_cis_*, 1H), 4.38 (m, OC*H*_2_CH_2_, 2H), 4.34 (dd, ^3^*J*_H,F_= 49.3 Hz, ^2^*J*_H,H_ =3.0 Hz, CFC*H*_2_,H_trans_, 1H), 4.19 (m, OCH_2_C*H*_2_, 2H), 3.97 (dt, ^3^*J*_H,H_= 8.8 Hz, ^3^*J*_H,H_ = 5.7 Hz, NHC*H*, 1H), 2.62 (m, ^3^*J*_H,F_ = 20.5 Hz, ^3^*J*_H,H_ = 5.7 Hz, ^2^*J*_H,H_ =1.2 Hz, NHCHC*H*_2_, 2H), 2.46 (s, SO_2_PhC*H*_3_, 3H), 1.30 (s, C(C*H*_3_)_3_, 9H).^13^C NMR (75 MHz, CDCl_3_): δ = 169.2 (*C*O), 160.9 (^1^*J*_C,F_ = 257.1 Hz, *C*FCH_2_), 161.4 (qAr*C*OCH_2_CH_2_OSO_2_), 145.2 (qAr*C*CH_3_), 132.6 (OSO_2_*C*Ar), 132.1 (qAr*C*SO_2_), 129.9 (Ar*C*H), 129.4 (Ar*C*H), 128.0 (Ar*C*H), 114.6 (Ar*C*H), 94.2 (d, ^2^*J*_C,F_ = 18.8 Hz, CF*C*H_2_), 83.2 (*C*(CH_3_)_3_), 67.5 (O*C*H_2_CH_2_), 65.7 (OCH_2_*C*H_2_), 53.2 (NH*C*H), 36.4 (d, ^2^*J*_C,F_ = 27.4 Hz, NHCH*C*H_2_CF), 27.7 (C(*C*H_3_)_3_), 21.7 (SO_2_Ph*C*H_3_). ^19^F NMR (282 MHz, CDCl_3_): δ = -95.63 (ddt, ^3^*J*_H,F_= 49.3 Hz, ^3^*J*_H,F_ = 20.6 Hz, ^3^*J*_H,F_ = 17.0 Hz, 1F). MS-ES(+)-EM: *m/z* = 566.1281 [(M+Na)^+^] calcd for C_24_H_30_FNO_8_S_2_Na^+^: 566.1289.

*3.2. tert-Butyl (2S)-2-{N-benzyl-4-[2-(tosyloxy)ethoxy]phenylsulfonamido}-4-fluoropent-4-enoate ((S)-****6b****).* To a stirred solution of *tert*-butyl (2*S*)-4-fluoro-2-{4-[2-(tosyloxy)ethoxy]phenylsulfonamido}pent-4-enoate ((*S*)-**5b**, 1.60 g, 2.94 mmol) in DMF (50 mL) benzyl bromide (1.1 eq., 3.24 mmol, 0.55 g) and K_2_CO_3_ (10 eq., 4.05 g, 29.4 mmol) were added and the solution was stirred at room temperature over 2 d. The reaction mixture was diluted with water (200 mL) and extracted with ethyl acetate (3 × 100 mL). The combined organic layer was washed with brine and dried over MgSO_4_. After removing the solvent under reduced pressure column chromatographic purification (silica gel, cyclohexane/ethyl acetate 2:1) gave the carbonic acid ester as a colorlesswaxy oil. Yield:1.38 g (74%). ^1^H NMR (300 MHz, CDCl_3_): δ = 7.81 (d, ^3^*J*_H,H_= 8.3 Hz, Ar*H*, 2H), 7.78 (d, ^3^*J*_H,H_ = 9.0 Hz, Ar*H*, 2H), 7.35 (d, ^3^*J*_H,H_= 8.0 Hz, Ar*H*, 2H), 7.33 – 7.22 (m, Ar*H*, 5H), 6.84 (d, ^3^*J*_H,H_= 9.0 Hz, Ar*H*, 2H), 4.63 (d, ^2^*J*_H,H_= 15.8 Hz, NC*H*_2_, 1H), 4.55 (t, ^3^*J*_H,H_ = 7.2 Hz, NC*H*, 1H), 4.53 (dd, ^3^*J*_H,F_ = 17.2 Hz, ^2^J_H,H_ = 3.0 Hz, CFC*H*_2_,H*_cis_*, 1H), 4.39 (m, OC*H*_2_CH_2_, 2H), 4.28 (d, ^2^*J*_H,H_= 15.8 Hz, NC*H*_2_, 1H), 4.23 – 4.17 (m, OCH_2_C*H*_2_, 2H), 4.13 (dd, ^3^*J*_H,F_= 49.7 Hz, ^2^*J*_H,H_ 3.0 Hz, CFC*H*_2_, H_trans_, 1H), 2.70 (m, ^3^*J*_H,F_ = 15.3 Hz, ^3^J_H,H_ = 6.9 Hz, NCHC*H*_2_, 1H), 2.54– 2.34 (m, NCHC*H*_2_, 1H), 2.45 (s, SO_2_PhC*H*_3_, 3H), 1.36 (s, C(C*H*_3_)_3_, 9H). ^13^C NMR (75 MHz, CDCl_3_): δ = 168.5 (*C*O), 160.9 (d, ^1^*J*_C,F_ = 256.4 Hz, *C*FCH_2_), 161.2 (qAr*C*OCH_2_CH_2_OSO_2_), 145.2 (qAr*C*CH_3_), 136.6 (OSO_2_*C*Ar), 132.7 (qAr*C*CH_2_N), 132.6 (qAr*C*SO_2_), 129.9 (Ar*C*H), 129.8 (Ar*C*H), 128.5 (Ar*C*H), 128.4 (Ar*C*H), 128.0 (Ar*C*H), 127.8 (Ar*C*H), 114.5 (Ar*C*H), 93.1 (d, ^2^*J*_C,F_ = 19.1 Hz, CF*C*H_2_), 82.6 (*C*(CH_3_)_3_), 67.6 (O*C*H_2_CH_2_), 65.7 (OCH_2_*C*H_2_), 57.6 (N*C*H), 50.0 (N*C*H_2_), 34.0 (d, ^2^*J*_C,F_ = 27.9 Hz, NCH*C*H_2_CF), 27.8 (C(*C*H_3_)_3_), 21.6 (SO_2_Ar*C*H_3_).^19^F NMR (282 MHz, CDCl_3_): δ = -95.63 (m, ^3^*J*_H,F_= 49.3 Hz, ^3^*J*_H,F_ = 20.6 Hz, ^3^*J*_H,F_ = 17.0 Hz, 1F). MS-ES(+)-EM: *m/z* = 566.1281 [(M+Na)^+^] calcd for C_24_H_30_FNO_8_S_2_Na^+^: 566.1289.

*3.3. (2S)-2-{N-benzyl-4-[2-(tosyloxy)ethoxy]phenylsulfonamido}-4-fluoropent-4-enoic acid ((S)-****7b****).* (*S*)-*tert*-Butyl 2-{*N*-benzyl-4-[2-(tosyloxy)ethoxy]phenylsulfonamido}-4-fluoropent-4-enoate ((*S*)-**6b**, 1.38 g, 2.17 mmol) was dissolved in CH_3_CN (10 mL). KSF-clay (450 mg) was added and the mixture was refluxed for 3 h. The mixture was filtrated, washed with ethyl acetate (5 mL) and dried over Na_2_SO_4_. The solvent was removed under reduced pressure to give the colorless waxy product. Yield: 0.99 g (79%). ^1^H NMR (300 MHz, CDCl_3_): δ = 7.81 (d, ^3^*J*_H,H_= 8.9 Hz, Ar*H*, 2H), 7.80 (d, ^3^*J*_H,H_ = 8.4 Hz, Ar*H*, 2H), 7.35 (d, ^3^*J*_H,H_= 8.0 Hz, Ar*H*, 2H), 7.31 – 7.22 (br s, Ar*H*, 5H), 6.88 (d, ^3^*J*_H,H_= 8.9 Hz, Ar*H*, 2H), 4.53 (d, ^2^*J*_H,H_= 15.1 Hz, NC*H*_2_, 1H), 4.55 (t, ^3^*J*_H,H_= 7.2 Hz, NC*H*, 1H), 4.57 (dd, ^3^*J*_H,F_ = 17.1 Hz, ^2^J_H,H_ = 3.2 Hz, CFC*H*_2_*,* H*_cis_*, 1H), 4.41 – 4.32 (m, OC*H*_2_CH_2_, 2H), 4.33 (d, ^2^*J*_H,H_= 15.8 Hz, NC*H*_2_, 1H), 4.21 (dd, ^3^*J*_H,F_= 50.2 Hz, ^2^*J*_H,H_ 3.3 Hz, CFC*H*_2_*,* H_trans_, 1H), 4.28 – 4.20 (m, OCH_2_C*H*_2_, 2H), 2.99 – 2.71 (m, NCHC*H*_2_, 1H), 2.62 (ddd, ^3^*J*_H,F_= 23.4 Hz, ^2^*J*_H,H_ = 15.3 Hz, ^3^*J*_H,H_ = 8.4 Hz, NCHC*H*_2_, 1H), 2.45 (s, SO_2_PhC*H*_3_, 3H). ^13^C NMR (75 MHz, CDCl_3_): δ = 173.5 (*C*OOH), 161.4 (d, ^1^*J*_C,F_ = 256.0 Hz, *C*FCH_2_), 161.5 (qAr*C*OCH_2_CH_2_OSO_2_), 145.2 (qAr*C*CH_3_), 135.6 (OSO_2_*C*Ar), 132.5 (qAr*C*CH_2_N), 131.8 (qAr*C*SO_2_), 130.0 (Ar*C*H), 129.9 (Ar*C*H), 128.7 (Ar*C*H), 128.5 (Ar*C*H), 128.1 (Ar*C*H), 128.0 (Ar*C*H), 114.7 (Ar*C*H), 93.8 (d, ^2^*J*_C,F_ = 18.9 Hz, CF*C*H_2_), 67.8 (O*C*H_2_CH_2_), 65.7 (OCH_2_*C*H_2_), 56.3 (N*C*H), 50.5 (N*C*H_2_), 33.4 (d, ^2^*J*_C,F_ = 28.1 Hz, NCH*C*H_2_CF), 21.7 (SO_2_Ar*C*H_3_). ^19^F NMR (282 MHz, CDCl_3_): δ = -97.93 (dddd, ^3^*J*_H,F_= 49.9 Hz, ^3^*J*_H,F_ = 22.6 Hz, ^3^*J*_H,F_ = 17.1 Hz, ^4^*J*_H,F_ = 13.9 Hz, 1F). MS-ES(+)-EM: *m/z* = 600.1128 [(M+Na)^+^] calcd for C_27_H_28_FNO_8_S_2_Na^+^: 600.1133.

*3.4. 2-{4-[N-benzyl-N-((2S)-4-fluoro-1-oxo-1-{[(tetrahydro-2H-pyran-2-yl)oxy]amino}-pent-4-en-2-yl)sulfamoyl]phenoxy}ethyl 4-methylbenzenesulfonate ((S)-****8b****).* To a solution of (*S*)-2-(*N*-benzyl-4-(2-(tosyloxy)ethoxy)phenylsulfonamido)-4-fluoropent-4-enoic acid (**(S)-7b**, 1.10 g, 1.90 mmol) in DMF (9 mL) HOBT (1.2 eq., 0.31 g, 2.29 mmol), NMM (3 eq., 0.58 g, 0.63 mL), *O*-tetrahydro-2-*H*-pyran-2-yl-hydroxylamine (3.1 eq., 0.69 g, 5.89 mmol) and EDC (1.4 eq., 0.51 g, 2.66 mmol) were added. After stirring at room temperature overnight the reaction mixture was diluted with water (100 mL) and extracted with ethyl acetate (3 × 50 mL). The combined organic phases were washed successively with water, 5% aqueous KHSO_4_, saturated aqueous NaHCO_3_ and brine (100 mL) and dried over magnesiumsulfate. After removing the solvent under reduced pressure column chromatographic purification (silica gel, cyclohexane / ethyl acetate, 2:1) yielded the mixture of diastereomeric THP-protected hydroxamic acids as a colorless waxy oil. Yield: 0.78 g (61%). ^1^H NMR (300 MHz, CDCl_3_): δ = 9.09 (s, N*H*, 1H), 9.07 (s, N*H*, 1H), 7.82 (d, ^3^*J*_H,H_= 8.3 Hz, Ar*H*, 4H), 7.72 (d, ^3^*J*_H,H_ = 8.9 Hz, Ar*H*, 2H), 7.68 (d, ^3^*J*_H,H_ = 9.0 Hz, Ar*H*, 2H), 7.36 (d, ^3^*J*_H,H_= 8.0 Hz, Ar*H*, 4H), 7.33 – 7.21 (m, Ar*H*, 10H), 6.85 (d, ^3^*J*_H,H_= 9.0 Hz, Ar*H*, 2H), ), 6.83 (d, ^3^*J*_H,H_= 9.0 Hz, Ar*H*, 2H), 4.90 (m, NHOC*H*O, 1H), 4.68 (m, NHOC*H*O, 1H), 4.64 (d, ^2^*J*_H,H_= 15.9 Hz, NC*H*_2_, 1H), 4.52 – 4.43 (m, NC*H*, CFC*H*_2_*,* H*_cis_*, ^2^*J*_H,H_ = 2.9 Hz, 1.5H), 4.52 – 4.43 (m, OC*H*_2_CH_2_, CFC*H*_2_*,* H*_cis_*, ^2^*J*_H,H_ = 3.0 Hz, 2.5H), 4.46 (d, ^2^*J*_H,H_= 15.0 Hz, NC*H*_2_, 1H), 4.25 – 4.19 (m, OCH_2_C*H*_2_, 2H), 4.10 (dd, ^3^*J*_H,F_= 50.0 Hz, ^2^*J*_H,H_ 3.1 Hz, CFC*H*_2_*,* H_trans_, 1H), 3.98 – 3.83 (m, NHOCHOC*H*_2_, 2H), 3.74 – 3.58 (m, NHOCHOC*H*_2_, 2H), 2.93 – 2.61 (m, NCHC*H*_2_, 1H), 2.46 (s, SO_2_PhC*H*_3_, 3H), 2.44 – 2.23 (m, NCHC*H*_2_, 1H), 1.86 – 1.48 (m, THP-C*H*_2_, 6H). ^13^C NMR (75 MHz, CDCl_3_): δ = 165.75 (*C*ONH), 165.67 (*C*ONH), 161.6 (qAr*C*OCH_2_CH_2_OSO_2_), 161.2 (d, ^1^*J*_C,F_ = 255.7 Hz, *C*FCH_2_), 145.2 (qAr*C*CH_3_), 136.3 (OSO_2_*C*Ar), 132.6 (qAr*C*CH_2_N), 131.7 (qAr*C*SO_2_), 129.9 (Ar*C*H), 129.8 (Ar*C*H), 128.81 (Ar*C*H), 128.76 (Ar*C*H), 128.57 (Ar*C*H‘), 128.52 (Ar*C*H), 128.0 (Ar*C*H), 114.70 (Ar*C*H), 114.67 (Ar*C*H), 101.77 (NHO*C*HO), 101.76 (NHO*C*HO), 93.8 (d, ^2^*J*_C,F_ = 18.9 Hz, CF*C*H_2_), 67.5 (O*C*H_2_CH_2_), 65.8 (OCH_2_*C*H_2_), 62.1 (NHOCHO*C*H_2_), 54.5 (N*C*H), 48.7 (N*C*H_2_), 48.5 (N*C*H_2_), 33.4 (d, ^2^*J*_C,F_ = 28.1 Hz, NCH*C*H_2_CF), 27.7 (NHOCH*C*H_2_), 24.9 (CH_2_), 21.7 (SO_2_Ar*C*H_3_), 18.2 (NHOCHCH_2_*C*H_2_).^19^F NMR (282 MHz, CDCl_3_): δ =
-97.56 (m), -97.82 (m,^3^*J*_H,F_= 50.1 Hz, ^3^*J*_H,F_ = 37.1 Hz, ^3^*J*_H,F_ = 17.9 Hz, 1F). MS-ES(+)-EM: *m/z* = 669.1809 [(M+Na)^+^] calcd. for C_32_H_37_FN_2_O_9_S_2_Na^+^: 669.1817.

*3.5. (S)-2-(4-{N-benzyl-N-[4-fluoro-1-(hydroxyamino)-1-oxopent-4-en-2-yl]sulfamoyl}-phenoxy)ethyl-4-methylbenzenesulfonate ((S)-****9b****).* The reaction was carried out in a 0.30 mmol scale with 2-{4-[*N*-benzyl-*N*-((2*S*)-4-fluoro-1-oxo-1-{[(tetrahydro-2*H*-pyran-2-yl)oxy]-amino}pent-4-en-2-yl)sulfamoyl]phenoxy}ethyl-4-methylbenzene-sulfonate ((*S*)-**8b**). After column chromatography (silica gel, cyclohexane / ethyl acetate, 1:1) a light brown wax was obtained. Yield: 75 mg (53%). ^1^H NMR (300 MHz, CDCl_3_): δ = 9.24 (br s, 1-OH), 7.81 (d, ^3^*J*_H,H_= 8.3 Hz, Ar*H*, 2H), 7.67 (d, ^3^*J*_H,H_ = 8.8 Hz, Ar*H*, 2H), 7.35 (d, ^3^*J*_H,H_= 8.0 Hz, Ar*H*, 2H), 7.32 – 7.27 (br s, Ar*H*, 5H), 6.83 (d, ^3^*J*_H,H_= 8.8 Hz, Ar*H*, 2H), 4.53 (d, ^2^*J*_H,H_= 15.6 Hz, NC*H*_2_, 1H), 4.51 (t, ^3^*J*_H,H_ = 8.3 Hz, NCH, 1H), 4.60 – 4.46 (m, CFC*H*_2_, H*_cis_*, 1H), 4.46 – 4.30 (m, OC*H*_2_CH_2_, NC*H*_2_, 3H), 4.24 – 4.17 (m, OCH_2_C*H*_2_, 2H), 4.08 (dd, ^3^*J*_H,F_= 49.7 Hz, ^2^*J*_H,H_ 3.0 Hz, CFC*H*_2_, H_trans_, 1H), 2.85 – 2.64 (m, NCHC*H*_2_, 1H), 2.46 – 2.24 (m, NCHC*H*_2_, 1H), 2.45 (s, SO_2_ArC*H*_3_, 3H). ^13^C NMR (75 MHz, CDCl_3_): δ = 166.4 (*C*ONH), 161.6 (qAr*C*OCH_2_CH_2_OSO_2_), 161.0 (d, ^1^*J*_C,F_ = 256.3 Hz, *C*FCH_2_), 145.2 (qAr*C*CH_3_), 136.1 (OSO_2_*C*Ar), 132.6 (qAr*C*CH_2_N), 131.4 (qAr*C*SO_2_), 129.9 (Ar*C*H), 129.6 (Ar*C*H), 128.57 (Ar*C*H), 128.56 (Ar*C*H),127.97 (Ar*C*H), 127.93 (Ar*C*H), 114.7 (Ar*C*H), 93.8 (d, ^2^*J*_C,F_ = 18.8 Hz, CF*C*H_2_), 67.6 (O*C*H_2_CH_2_), 65.7 (O*C*H_2_CH_2_), 54.2 (N*C*H), 48.7 (N*C*H_2_), 31.9 (d, ^2^*J*_C,F_ = 27.5 Hz, NCH*C*H_2_CF), 21.6 (SO_2_Ar*C*H_3_). ^19^F NMR (282 MHz, CDCl_3_): δ = -97.59 (ddd, ^3^*J*_H,F_= 50.1 Hz, ^3^*J*_H,F_ = 37.1 Hz, ^3^*J*_H,F_ = 17.4 Hz,1 F). MS-ES(+)-EM: *m/z* = 615.1253 [(M+Na)^+^] calcd for C_27_H_29_FN2O_8_S_2_Na^+^: 615.1242. HPLC: t*_R_* = 9.95 min (100%).

**References**

Laue KW, Kröger S, Wegelius E, Haufe G. Stereoselective synthesis of ɣ-fluorinated α-amino acids using 2-hydroxy-3-pinanone as an auxiliary. Eur J Org Chem. 2000;3737–3743.

Wagner S, Faust A, Breyholz HJ, Schober O, Schäfers M, Kopka K. The MMP inhibitor (*R*)-[*N*-benzyl-4-(2-[^18^F]-fluoroethoxy)phenylsulfonamido-*N*-hydroxy-3]methylbutanamide: improved precursor synthesis and fully automated radiosynthesis. Appl Radioat Isot. 2011;69:862–868.

**Figure 1**: ^1^H NMR of (*S*)-**5a**

**Figure 2**: ^1^H NMR of (*S*)-**6a** (contains ethyl acetate)

**Figure 3**: ^1^H NMR of (*S*)-**7a** in acetonitrile-d_3_

**Figure 4**: ^1^H NMR of (*S*)-**8a** (contains ethyl acetate)

**Figure 5**: ^1^H NMR of (*S*)-**9a** (contains ethyl acetate)

**Figure 6**: ^1^H NMR of (*R*)-**5a**

**Figure 7**: ^1^H NMR of (*R*)-**6a** (contains ethyl acetate)

**Figure 8**: ^1^H NMR of (*R*)-**7a** in acetonitrile-d_3_

**Figure 9**: ^1^H NMR of (*R*)-**8a** (contains ethyl acetate)

**Figure 10**: ^1^H NMR of (*R*)-**9a** (contains ethyl acetate)

**Figure 11**: ^1^H NMR of (*S*)-**5b**

**Figure 12**: ^1^H NMR of (*S*)-**6b** (contains ethyl acetate)

**Figure 13**: ^1^H NMR of (*S*)-**7b** (contains ethyl acetate)

**Figure 14**: ^1^H NMR of (*S*)-**8b** (contains ethyl acetate)

**Figure 15**: ^1^H NMR of (*S*)-**9b** (contains ethyl acetate)


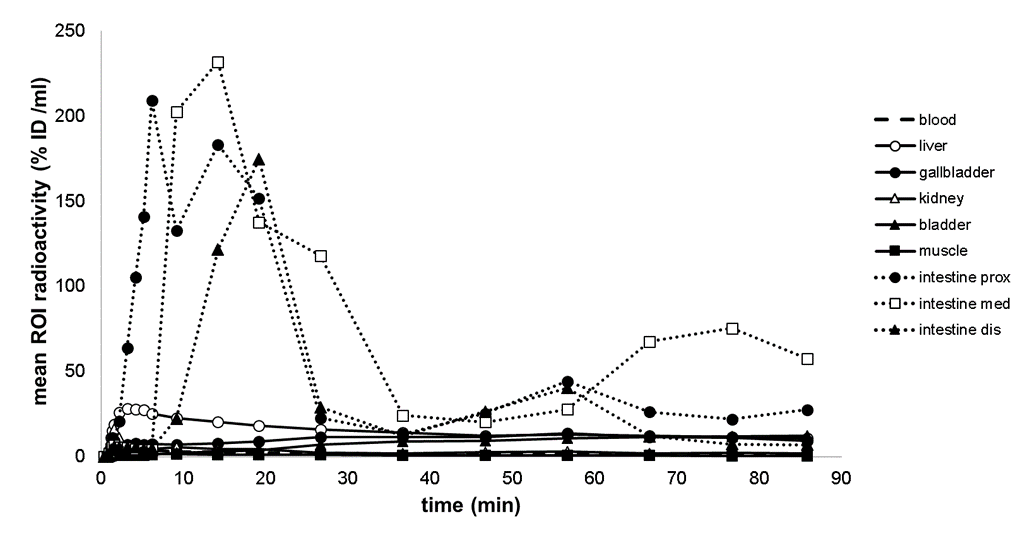


**Figure 16**: *In vivo* biodistribution of radioactivity in an adult C57/Bl6 mouse after intravenous injection of (*S*)-[^18^F]**9a**. Time-activity concentration curves illustrate intestinal transport of hepatobiliary eliminated (*S*)-[^18^F]**9a**. ROI: regions of interests. % ID: percentage injected dose.
